# Knowledge, attitudes, and practices related to TB among the general population of Ethiopia: Findings from a national cross-sectional survey

**DOI:** 10.1371/journal.pone.0224196

**Published:** 2019-10-28

**Authors:** Daniel G. Datiko, Dereje Habte, Degu Jerene, Pedro Suarez

**Affiliations:** 1 Challenge TB and Management Sciences for Health, Addis Ababa, Ethiopia; 2 Management Sciences for Health, Infectious Diseases Cluster, Arlington, Virginia, United States of America; Institute of Economic Growth, INDIA

## Abstract

**Introduction:**

Ethiopia is among the high-burden countries for tuberculosis (TB), TB/HIV, and drug-resistant TB. The aim of this nationwide study was to better understand TB-related knowledge, attitudes, and practices (KAPs) and generate evidence for policy and decision-making.

**Materials and methods:**

We conducted a cross-sectional TB KAP survey in seven regions and two city administrations of Ethiopia. Eighty *kebeles* (wards) and 40 health centers were randomly selected for the study. Using systematic sampling, 22 households and 11 TB patients were enrolled from each selected village and health center, respectively. Variables with a value of *p* = < 0.25 were included in the model for logistic regression analysis.

**Results:**

Of 3,503 participants, 884 (24.4%), 836 (24.1%), and 1,783 (51.5%) were TB patients, families of TB patients, and the general population, respectively. The mean age was 34.3 years, and 50% were women. Forty-six percent were heads of households, 32.1% were illiterate, 20.3% were farmers, and 19.8% were from the lowest quintile. The majority (95.5%) had heard about TB, but only 25.8% knew that TB is caused by bacteria. Cough or sneezing was reported as the commonest means of TB transmission. The majority (85.3%) knew that TB could be cured. Men, better-educated people, and TB patients and their families have higher knowledge scores. Of 2,483 participants, 96% reported that they would go to public health facilities if they developed TB symptoms.

**Discussion:**

Most Ethiopians have a high level of awareness about TB and seek care in public health facilities, and communities are generally supportive. Inadequate knowledge about TB transmission, limited engagement of community health workers, and low preference for using community health workers were the key challenges.

**Conclusions:**

Given misconceptions about TB’s causes, low preference for use of community health workers, and inadequate engagement, targeted health education interventions are required to improve TB services.

## Introduction

Worldwide, tuberculosis (TB) is the leading cause of death from a single infectious agent. In 2017 about 10 million TB cases were estimated to occur, a third of them were missed, and about 1.6 million died in the same year [1]. TB is inequitably distributed and clustered among disadvantaged and socioeconomically deprived population groups [2–5]. TB is primarily a disease of the poor and its magnitude is high in socially disadvantaged populations or people residing in poor living condition, which are characterized by lack of education, poor housing, inadequate nutrition, overcrowding, and socioeconomic factors. Lack of awareness prevails in populations living in poor conditions, which leads to delay in health care-seeking due to lack of knowledge about the symptoms of TB and of prevention measures. Lack of awareness, in turn, leads to further transmission of the disease and poor treatment outcomes.

The decline of TB in developed countries with improved living conditions [6–8] indicates that poor living conditions, as reflected by lack of awareness, stigma, poor health care–seeking behavior, and deficient health systems, favor TB transmission and occurrence of disease [9–11]. In addition, distance, cost, and sociocultural barriers limit care seeking [12–14].

Studies have shown that awareness of or knowledge about TB and the availability of patient-centered services in settings with high burdens of human immunodeficiency virus (HIV) and TB is inadequate [15–17]. Generally, TB-related knowledge and attitudes vary across countries, ranging from an understanding of its infectious cause to the belief that its cause is the evil eye, and from supportive to highly stigmatized views toward the disease and patients. Adequate knowledge and positive attitudes about TB patients are expected to contribute to improved health care–seeking behavior. However, awareness about TB and the availability of services are often found to be suboptimal among underprivileged social groups, and illiterate, inaccessible, rural, and impoverished communities [18–22].

Ethiopia is among high-burden TB, TB-HIV, and drug-resistant countries. It has implemented TB programs for decades and rapidly decentralized TB services. However, the National TB Program (NTP) continues to miss about a third of estimated TB cases. This could be due to lack of awareness about TB, lack of access, poverty, and TB-associated stigma, as indicated by studies conducted in some parts of the country [23–25]. Therefore, understanding knowledge, attitudes, and practices (KAPs) related to TB and their underlying causes is important to design national responses to improve TB services in the communities of Ethiopia.

The NTP of Ethiopia has decentralized services to the community by deploying health extension workers (HEWs), who deliver preventive and promotive health services, including health education, to improve community awareness and enhance service delivery [26]. Although there is no direct measurement of the results of such efforts, evidence shows improvements in the level of awareness of or knowledge about TB [16, 23, 27–29]. However, these lacked the depth of information required to understand KAPs, did not address sub-population groups, and were limited to smaller geographic areas. The aim of this study was to better understand national TB- related KAPs in Ethiopia and generate evidence for policy and decision-making.

## Materials and methods

### Study setting, population, and design

This TB KAP survey was conducted in seven regions and two city administrations of Ethiopia, covering 92% of the national population. Ethiopian Somali and Afar regions were excluded from the survey because they were not directly supported by the Challenge TB Project. Ethiopia is the third most populous country in Africa, with a population of more than 100 million people, of whom 85% live in rural areas. Currently, 256 hospitals and 3,390 health centers provide TB services, and over 16,000 health posts deliver community-based TB services in the country.

This was a cross-sectional population-based survey conducted from October to November 2017. A single population proportion formula was used to estimate sample size based on multiple indicators from the Ethiopia Demographic and Health Survey 2011 report [7]. For the other indicators where previous nationwide reports were unavailable, we used a percentage of 50% to obtain the maximum sample size. We used a design effect of 2 to adjust for the multistage cluster sampling and added 10% to adjust for non-response (S1 Table). The estimated sample was 3,463 in total and shown by regions (S8 Table).

**Fig 1:**
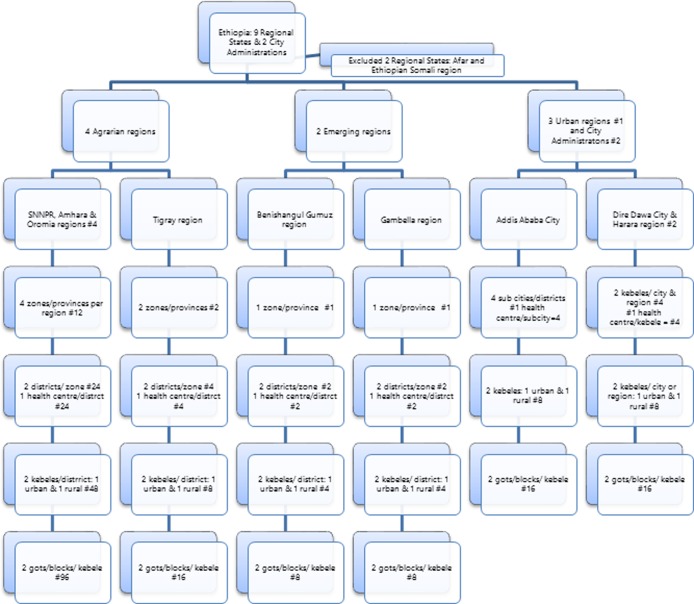
Study sites for the knowledge attitude and practice related to tuberculosis in Ethiopia.

Sixteen zones (provinces) and four sub-cities were selected from the regions and city administrations included in the study. From each zone or sub-city, two districts or *kebeles* (wards) were randomly selected. From each district, one rural and one urban kebele were identified for the study. The total number of kebeles included in the study was 80, which were divided into clusters as the final study unit. Households were identified by systematic sampling, and 22 participants were enrolled from the selected households in the clusters. Forty health centers were selected from districts of the regions, sub-cities, and kebeles of urban regions or cities (Fig 1) shows the description of the study site selection for the KAP survey in Ethiopia. Once we had selected the health centers and villages for study, we interviewed 11 TB patients from each health center and their families. The general population groups were selected from randomly selected blocks or clusters in urban and rural villages, respectively. Of these, 22 households were selected using a systematic sampling technique. From the households, a household member at least 18 years old who had lived in the house for at least 6 months was selected by lottery. We selected male and female participants alternately to ensure gender balance. If no one was home at the selected household during the day of data collection, it was replaced by the adjacent household.

### Data collection tools, data analysis, and quality assurance

We collected KAP data from the sub-populations using data collection tool (S2 Table) but did not conduct any interviews of TB patients related to attitudes. Attitude-related interviews were conducted for the general population and families of TB patients, representing 2,619 participants. Under each category of the data collection tool, there were many variables for KAPs, which we categorized to measure the outcomes. Under educational status, those who attended but did not complete primary school were classified as “read and write only,” while those who completed education until grade six were classified as having a primary school education.

We identified the total number of interview questions used to assess the knowledge of the study participants and the total number of expected correct answers. We calculated knowledge scores using the mean of the number of correct answers by the study participants as a cutoff point to categorize high or low knowledge scores. The study participants who answered above the mean score were classified as having a high score, while those who scored below the mean were classified as having a low knowledge score.

The national wealth index was constructed using the World Health Organization (WHO) tool for KAP surveys [30]. Wealth-related variables were initially constructed for rural and urban populations, and later we constructed a common wealth index using variables that were considered common for both rural and urban areas. Finally, both the rural and urban wealth index regression coefficients were mapped into the common wealth index, resulting in a composite “national” wealth index, which was categorized into quintiles.

We used pretested semi structured questionnaires, adopted from the WHO guide, to collect quantitative data (30). Tablets were used to collect data using the Web-based platform of Census and Survey Processing System (CSPro) software.

Data extracted from this platform were exported to SPSS version 25.0 (IBM SPSS Statistics, 2019) for analysis. We calculated composite knowledge scores using knowledge variables and used mean knowledge scores to dichotomize results into high and low scores. Variables associated with the outcome variable with a *p* value less than 0.25 were included in the multivariate regression model. A *p* value less than 0.05 was considered to be statistically significant.

We trained experienced data collectors and supervisors who speak local languages. The questionnaires were prepared in English, translated into regional languages, and back-translated to check for translation accuracy. Supervisors conducted household and random data checks. The data manager, a CSPro expert, centrally checked for data quality. A central research team supervised the data collection process.

### Ethical considerations

The National Research Ethics Review Committee of the Ministry of Science and Technology approved the study. Oral informed consent was requested, as some of the study participants were illiterate and from rural communities, which makes obtaining written consent problematic. The Federal Ministry of Health provided a letter of support to conduct the study. Oral informed consent was obtained from the study participants and documented (S1 File).

## Results

### Sociodemographic characteristics of the study participants

We enrolled 3,503 study participants. Of these, 884 (24.4%), 836 (24.1%), and 1,783 (51.5%) were TB patients, families of TB patients and general population respectively. The mean age and standard deviation were 34.3 + 12.9 years for both sexes, 34.9 + 13.2 for men and 33.8 + 12.5 for women. Fifty per cent of the study participants were in the age range of 18–30 years and 50% were women. Forty-six per cent (1,594) were heads of households, 62.2% were married, 32.1% were illiterate, 20.3% were farmers, and 19.8% were from lowest quintile (Table 1).

**Table 1:** Sociodemographic characteristics of the study participants, 2017.

Variables	Variable Categories	Frequency	Percent
Population type	General population	1,783	51.5
	TB patients	844	24.4
	Families of TB patients	836	24.1
Sex	Male	1,730	49.96
	Female	1,733	50.04
Age in years	18–30	1,732	50.0
	31–60	1,587	45.8
	> 60	144	4.2
Relationship to head of household	Head	1,594	46.0
	Spouse	1,034	29.9
	Son/daughter	648	18.7
	Other relative	165	4.8
	Non-relative	22	0.6
Marital status	Married	2,153	62.2
	Never married/never lived together	829	23.9
	Divorced/separated	255	7.4
	Widowed	202	5.8
	Living together	24	0.7
Educational Status	Not able to read and write	1,113	32.1
	Read and write only^1^	219	6.3
	Primary^2^	1,007	29.1
	Secondary	734	21.2
	Above secondary	390	11.3
Occupation	Employed	438	12.7
	Housewife	685	19.8
	Farmer	703	20.3
	Daily laborer	362	10.4
	Trader	587	17.0
	Student	329	9.5
	No job/dependent	263	7.6
	Housemaid	75	2.2
	Others	21	0.6
Wealth quintile	Lowest	684	19.8
	Second	909	26.2
	Third	820	23.7
	Fourth	643	18.6
	Highest	407	11.8

1 Those who went to school but did not complete the primary level of education

2 Those who have completed education through grade six

### Knowledge about TB: Sources of information, cause, transmission, and prevention

Most of the population, 3,306 (95.5%) had heard about TB and 25.8% (986) knew that TB is caused by bacteria. However, 47% (1,626) of the participants did not know the cause of TB. Cough or sneezing was reported as a means of TB transmission by 70.4% (2,585) of the respondents. The commonest symptoms were cough in 85.5% (2,975), chest pain in 17.2% (596), fever in 17.1% (593), and other symptoms (weight loss, poor appetite, night sweats, blood in the sputum, shortness of breath, fatigue, or body swelling) in 67.6% (2,340) of participants.

Most of the study participants, 2,627 (75.9%), knew that anyone could get TB. Lung and bone were mentioned to be affected most by 80% (2,771) and 23.3% (807) of participants, respectively. Most of the study participants, 85.3% (2,953), knew that TB could be cured by taking medicine (Table 2).

**Table 2:** Sociodemographic characteristics of the study participants, 2017.

Variables (n = 3,463)	Responses	N	%
Have you ever heard of TB?	Yes	3,306	95.5
	No	157	4.5
Cause of TB	Bacteria	986	28.5
	Superstitions^1^	739	21.3
	Don’t know	1,626	47.0
How can a person get TB?	Coughing or sneezing	2,424	70.0
	Drinking raw milk	161	4.7
	Proximity^2^	2,141	61.8
	Don’t know	472	13.6
Who can be infected with TB?	Anyone	2,627	75.9
	HIV-infected people only	210	6.1
	Poor people only	531	15.3
	Poor behavior^3^	671	19.4
Body parts affected by TB	Lung	2,771	80.0
	Intestine	290	8.4
	Bone	807	23.3
	Lymph nodes^4^	272	7.9
	Others	122	3.5
	Don’t know	382	11.0
Symptoms of TB	Cough	2975	85.9
	Chest pain	596	17.2
	Fever	593	17.1
	Other constitutional symptoms^5^	2,340	67.6
	Don’t know	163	4.7
Is TB a preventable disease?	Yes	2,638	76.2
	No	243	7
	Don’t know	425	12.3
Prevention methods	Avoiding cough in front of people	1,873	54.1
	Safe disposal of sputum	852	24.60
	Ventilation of living room	608	17.6
	Avoiding close contact with TB patients	1,065	30.8
	Vaccination of children	170	4.9
	Others	285	8.2
	Don’t know	206	6.0
Can TB be cured?	Yes	3,078	88.9
	No	63	1.8
	Don’t know	165	4.8

^1^Evil eye, Satan, witchcraft, other causes

^2^ Sharing utensils/bed; touching a person with TB; through food or water; sexual contact with a person who has TB; mosquito bites; exposure to cold; others

^3^Only homeless people; only alcoholics; only drug users; only those who have been in prison; others

^4^ Refers to swelling around the neck, armpit, and inguinal areas that has lasted at least two weeks

^5^ Weight loss; poor appetite; night sweating; blood in the sputum; shortness of breath; fatigue; swelling; others

TB patients and their families had better knowledge about TB related to the body parts it affects, whether TB is curable or not, and how TB can be cured compared to the general population. However, knowledge about the causes and means of transmission among the three groups was low (S3 Table).

Most people, 668 (93.6%), of the population had heard about TB; 53.4% had heard from family, friends, neighbors, and colleagues. Television and radio contributed to 35.1% and 36.3%, respectively. Less than a third of the study population, 30.4% and 7.7%, had heard about TB from HEWs and Health Development Army (HDAs) respectively. The population that had heard from TV and radio varied by region (*p* < 0.05) but was smallest in Amhara Region. Family, friends, neighbors, and colleagues contributed more as a source of information about TB in Oromia and Amhara regions (*p* < 0.05) (S4 Table).

About 20.9% (349) of the general population, 25.3% (238) of families of TB patients, and 28.9% (238) of TB patients have heard about DR-TB. Three-fourths of the sub-population knew that it was often caused by irregular intake of anti-TB drugs. Twenty-four percent of the study participants responded that they had heard about DR-TB, and the figure was higher among TB patients and their families compared to the general population. Of those who had heard about DR-TB, at least 75% indicated irregular drug intake as the main reason for its development. Less was known about its dangerousness, transmission, and possibility of cure (S4 Table).

### Factors associated with knowledge

Men, better–educated people, and TB patients and their families had higher knowledge. Gambella and Oromia regions had higher knowledge than other regions, while Amhara Region had lower knowledge compared to other regions. Study participants from the lowest and second quintiles had lower knowledge. There was no significant difference whether the participants were from urban or rural kebeles (Table 3). Generally, knowledge scores were higher in families of TB patients and TB patients compared to the general population (S5–S7 Tables).

**Table 3:** Factors associated with knowledge about TB in Ethiopia, 2017.

Factors		COR	95% CI	AOR	95% CI
Sex	Female	1		1	
	Male	1.37	1.16–1.61	1.27	1.05–1.52
Education	Illiterate	1		1	
	Read and write only	1.66	1.19–2.33	1.62	1.13–2.31
	Primary	2.33	1.91–2.86	2.10	1.68–2.62
	Secondary	3.96	3.06–5.12	3.30	2.49–4.38
	Above secondary	7.91	5.14–12.17	5.88	3.70–9.34
Population type	General population	1		1	
	TB patients’ families	1.61	1.30–1.99	1.72	1.37–2.16
	TB patients	1.31	1.07–1.60	1.46	1.17–1.83
Wealth quintile	Lowest	1		1	
	Second	1.44	1.16–1.78	1.27	0.99–1.61
	Third	2.12	1.67–2.69	1.92	1.44–2.56
	Fourth	3.09	2.35–4.07	2.76	1.93–3.95
	Highest	7.38	4.79–11.39	5.56	3.32–9.31
Residence	Rural	1		1	
	Urban	1.78	1.51–2.10	0.90	0.72–1.11
Region	Amhara	1		1	
	SNNP	1.83	1.44–2.31	2.78	2.14–3.61
	Tigrai	3.32	2.36–4.68	4.08	2.87–5.88
	Benshangul Gumuz	2.45	1.61–3.73	3.51	2.25–5.49
	Gambella	3.40	2.07–5.59	5.91	3.49–9.98
	Addis Ababa	6.28	4.12–9.59	4.08	2.58–6.45
	Dire Dawa	2.27	1.51–3.42	1.79	1.15–2.80
	Harari	1.77	1.20–2.60	1.64	1.08–2.48
	Oromia	2.47	1.93–3.17	4.56	3.45–6.01

### Attitude about tuberculosis

A total of 2,619 participants (families of TB patients and the general population) were interviewed. Half of the respondents, 51% (1,270), thought that they could get TB in their lifetime. Sixty-six percent (1,634) reported that they would cope with TB, while 32% (786) reported that they would be afraid. About 5% reported that they would be surprised, ashamed, or embarrassed, or would feel sad or hopeless if they acquired TB (Table 4).

**Table 4:** Attitudes of study participants about tuberculosis.

Questions	Variables	Responses	Number	Percent
Do you think you could get TB?		No	1,213	49%
		Yes	1,270	51%
What would be your reaction if you acquired TB?	Cope with it	Yes	1,634	66%
		No	849	34%
	Fear	Yes	786	32%
		No	1,697	68%
	Surprise	Yes	117	5%
		No	2,366	95%
	Shame	Yes	72	3%
		No	2,411	97%
	Embarrassment	Yes	48	2%
		No	2,435	98%
	Sadness/hopelessness	Yes	115	5%
		No	2,368	95%
Whom will you inform if you get TB?	Doctor/health worker	Yes	2,065	83%
		No	418	17%
	Spouse	Yes	532	21%
		No	1,951	79%
	Parent	Yes	690	28%
		No	1,793	72%
	Children	Yes	245	10%
		No	2,238	90%
	Other family member	Yes	632	25%
		No	1,851	75%
	Close friend	Yes	381	15%
		No	2,102	85%
	No one	Yes	22	1%
		No	2,461	99%

### TB-related practices

Of 2,483 participants, 96% reported that they would go to public health facilities if they developed TB symptoms, while 13% preferred private facilities, 3% pharmacies, and 1% traditional healers. Of 2,463 respondents, 63% mentioned that they would go to health facilities immediately, while 30% would go in two weeks and 6% would go after two weeks.

We interviewed 1,668 study participants about practices. Of these, 197 (11.7%) had had TB symptoms during the study period. Of the 197, 67.5% visited public facilities, 10.7% visited pharmacies, and 17.3% did nothing. Among 210 respondents, those who had TB symptoms were advised by health care workers or community health extension workers to visit public and private facilities in 71.9% and 7.1% of cases, respectively. From the study participants who sought care, 5% contacted HEWs and HDAs for advice. Only 6 study participants (0.4%) informed HEWs and HDAs if they knew a person with TB symptoms. About a quarter (23.3%) of the population did nothing when they found presumptive TB cases in their community, while 3.2% (54) participated in presumptive TB case identification (Table 5).

**Table 5:** Practices related to TB among the general population in Ethiopia, 2017.

Variable		N	%
Action taken when experienced cough of at least 2 weeks		197	11.1
	Sought care from health institution	133	67.5
	Sought care from pharmacies	21	10.7
	Contacted HDA to get advice	5	2.5
	Contacted HEW to get advice	5	2.5
	Visited spiritual/traditional healer	7	3.6
	Did nothing	34	17.3
Action taken when encountered a person who had cough for at least 2 weeks		210	11.8
	Advised to seek care from public health institutions	151	8.5
	Advised to seek care from private health facilities	15	0.8
	Advised to seek care from pharmacies	10	0.6
	Informed the HDA to advise him/her	5	0.3
	Informed the HEW to advise him/her	1	0.1
	Advised to seek care from spiritual/traditional healer	4	0.2
	Did nothing	49	2.8
Involvement in TB prevention and control		54	3.0
	Referred family member to health facility	17	1.0
	Referred community member to health facility	26	1.5
	Involved in TB screening at community level	11	0.6
	Involved in tracing TB treatment defaulters	348	19.5
	Advised parents to get their infants vaccinated for TB	257	14.4
	Advised TB patients to take their drugs properly	141	7.9
	Served as TB treatment supporter	111	6.2
	Has family member with cough for 2 or more weeks	84	4.7
Action taken when family member had cough		111	6.7%
	Did nothing	27	1.5
	Took to public health facility	66	3.7
	Took to private health institution	12	0.7
	Took to pharmacy	3	0.2
	Took to spiritual/traditional healer	3	0.2

## Discussion

A high level of awareness about TB, supportive communities, and health care–seeking in public health facilities characterize the general population of Ethiopia. Most of the participants have heard about TB. However, inadequate knowledge about its transmission, limited engagement of community health workers, and low preference of the community for using community health workers were key challenges. To address them will require strengthening community-level interventions in Ethiopia. The findings also suggest the need for targeted health education interventions to close knowledge gaps and reach the most disadvantaged and affected communities.

A nationwide response to end TB requires adequate community knowledge about TB and its prevention and care [31]. We report higher knowledge about TB than other reports from Africa [17, 32–34] but lower knowledge than reports from Bangladesh, one of the high burden countries [35]. This could be due to difference in the study period, types of population groups studied, and existing health system [15]. The higher knowledge in Ethiopia could be explained by the increased access to primary health care created by the engagement of community health workers. There are also regional knowledge variations, which could be explained by the extent of community engagement and the level of regional capacity. The lower level of knowledge in Amhara Region compared to other regions of Ethiopia could be a result of lower engagement of the HEWs in TB work or sociocultural factors despite more than a decade of intensive support. Improving the engagement of HEWs in providing continued TB-related health education requires consideration, as recommended in a previous study from the region [27].

Several studies have shown that TB awareness is higher in urban communities than in rural communities, due to the accessibility of health services and better socioeconomic conditions [21, 36–38]. However, unlike many studies, this study did not find knowledge differences between rural and urban communities of Ethiopia, which could be due to the community-based health extension program the conduct health education sessions in the community [25, 39]. However, we report that HEWs were contacted by only 5% of those sought care. This finding suggests a need to strengthen support for the community health program [40]. The results about the contribution of the HEWs may seem contradictory. Their effectiveness is affected by the level of support they receive, the strength of the health system, and commitment of the HEWs and other motivational factors, which vary across regions and limit the contributions of the HEWs. In the southern region of Ethiopia, where HEWs are actively engaged in TB prevention and control, however, they have significantly contributed to increased community knowledge about TB [41–43].

The main sources of information about TB were close relatives [8, 16] followed by mass media, radio, and television [44]. This indicates that the tradition of sharing health information within a society could be exploited as a means to reach the community and to design interventions to enhance community awareness. This could be an opportunity in countries where community health workers live and work in their communities.

Sub-population analysis showed that the general population has a lower level of knowledge about TB compared to TB patients and their families (S3 Table). This could be due to health education and counseling services provided by health workers and the presence of a TB patient in the household. Therefore, health education should be geared to raise the awareness of the general population using the health education media most accessible to the community (S4 Table).

Knowledge scores among TB patients were similar to those of their families and the general population. A patient-level study in Ethiopia showed that knowledge about TB among TB patients is just as low as in the general population [45]. However, knowledge varied by setting, socioeconomic condition, stigma, sex, and educational status [18, 28, 45, 46]. Men had better knowledge about TB compared to women. This could be due to access to better socioeconomic conditions, such as education and wealth, which in turn increased their access to health information and care. In addition, urban residence affected the level of knowledge about TB.

The level of community awareness about TB shapes the perceptions of the community about TB and affects health care–seeking behavior, the type of support the patient receives from the household or community, adherence to treatment, and future engagement in TB prevention and control efforts [23, 36]. In our study, the community was supportive of TB patients within the household and helped patients to adhere to treatment. This could be due to the low prevalence of HIV, sociocultural values of communal living, and lower awareness about TB transmission in rural settings. Among the study participants interviewed, 76% were heads of households or their spouses. This might have contributed to the creation of supportive communities. Studies from urban areas, however, indicate that there are negative perceptions about TB due to high HIV prevalence and its associations [47]. Attitudes about TB and health care–seeking are shaped by educational status, knowledge, and socioeconomic conditions, as found in other studies [19. 37. 48].

The generally low knowledge about DR-TB among the study population will remain a great challenge for the NTP in the fight against increasing drug resistance. However, most of those who had heard about DR-TB responded that it is caused by irregular drug intake, which is important information to communicate to patients and their families to encourage adherence to treatment. The NTP needs to ensure the inclusion of health education about DR-TB in health education sessions at health facilities and in communities if prevention and control of DR-TB are to be successful.

Most of the study participants mentioned that they could cope with TB if they acquired it. Compared to the report from Nigeria that reported depression as high as 45% among patients and 13.4% among family members [49], we found only about 5% of people with reactions that included surprise, shame, embarrassment, sadness, or hopelessness. This could be due to the lack of awareness about the risk of acquiring TB, as 49% of our respondents did not know that anyone can get TB. However, our findings could be an underestimate, since the study was not designed to assess people for depression. The self-perceptions of the patients and perceptions of the community toward them, and the impact of those perceptions on TB prevention and care, warrant further study.

Adequate knowledge about TB, availability of affordable services, and reassuring community support increase the capacity of patients to disclose their medical condition, seek care, and adhere to treatment [50]. Moreover, a study from Malawi reported that patients were interested in disclosing their status if they would not be stigmatized [51]. However, community reactions that TB patients are inferior, should feel ashamed, and should be avoided could affect their ability to disclose their illness [52]. In our study, only 28% of the study participants reported that they would disclose their TB status to their parents, as opposed to higher disclosure rates reported from Nigeria (86%) and Ghana (68% among women and 75% among men) [53, 54]. We report the level of misconceptions about the cause of TB to be about 21% lower than in similar studies in Ethiopia, which reported misconceptions of about 50% and higher stigma [16, 55]. This could be due to low awareness of stigma associated with TB that affects the capacity to disclose and warrants further study.

The strength of this study was that it is the first national-level survey in Ethiopia that explored different population groups. One of the limitations of the study is that it did not include two pastoralist regions, which could limit its generalizability. However, similar communities were included from other regions to compensate for the missing information. Second, we did not study the attitudes of TB patients about TB, which limits the possibility of comparing them with those of other sub-populations in the study. The results might have been affected by the capacity of the study participants to understand and respond to the questionnaire. The respondents might also have given socially desirable answers, which could lead to overestimation of positive responses. Using trained and experienced data collectors who speak local languages and could explain the aim of study is likely to have reduced information bias.

## Conclusions

High community awareness, positive attitudes, and communities supportive of TB patients contribute to increased health care–seeking behavior. However, we found significant regional variations in the availability of adequate knowledge about the causative agent and means of transmission of TB, more so among underprivileged groups, the poor, those who are less educated, and women. These findings point to the need for targeted health education interventions to improve KAPs in the general population. The community generally has a positive attitude toward TB patients. However, people show limited interest in seeking care from community health workers. The NTP needs to address factors affecting the engagement of community health workers in TB prevention and control. Further studies are required to understand the reasons for the regional variations, to understand the extent of stigma and delay related to health care–seeking, and to improve the performance of the NTP.

## Supporting information

S1 Table sample size estimation. (PDF)

S2 Table Data collection tools. (PDF)

S3 Table Knowledge cause and risk of TB. (PDF)

S4 Table Knowledge source of information. (PDF)

S5 Table Factors of TB knowledge in the general population. (PDF)

S6 Table Factors TB knowledge in families of TB patients. (PDF)

S7 Table Factors TB knowledge in TB patients. (PDF)

S8 Table The study population by regions in Ethiopia. (PDF)

S1 File Informed consent. (PDF)
