# Supplementary material for: Knowledge, attitudes, and practices related to TB among the general population of Ethiopia: Findings from a national cross-sectional survey
Source: PLoS One. 2019 Oct 28;14(10):e0224196. doi: 10.1371/journal.pone.0224196 (PMC6816561; doi:10.1371/journal.pone.0224196)
Supplement: S1 Table — (PDF) [file pone.0224196.s001.pdf]

Supporting table 1 Sample size estimation for KAP study in Ethiopia

Domain	Indicator	Proportion (p)	Margin of error (w)	Confidence level	Design effect	Sample size
Knowledge	% of women in the GP who report that TB is spread through air by coughing	55.6%*	5%	95%	2	759
	% of men in the GP who report TB is spreads by coughing	65.0%*	5%	95%	2	699
	**Total**					**1,458**
	% of women in the GP who believe that TB can be cured	79.6%*	4%	95%	2	780
	% of men in the GP who believe that TB can be cured	89.3%*	4%	95%	2	459
	**Total**					1,**239**
	% of TB patients who have good knowledge on TB	50%	5%	95%	2	767
	% of family members of TB patients who have good knowledge on TB	50%	5%	95%	2	767
	% of women in the GP who would want a family member's TB kept secret	25.8%*	4%	95%	2	919
	% of men in the GP who would want a family member's TB kept secret	17.8%*	4%	95%	2	703
	**Total**					**1,622****
Attitu	% of TB patients who would not mind sharing other people that they've TB	50%	5%	95%	2	767**
	% of TB patients' family members who would not want a family member's TB status kept secret	50%	5%	95%	2	767
Practice	% of GP who have good practice related to TB services	50%	5%	95%	2	768**
	% of TB patients who have good practice related to TB services	50%	5%	95%	2	767
	% of family members of TB patients who have good practice related to TB services	50%	5%	95%	2	767

* Source-EDHS 2011 # GP - general population ** 10% was added for the sample size calculated to adjust for non-response rate.
