# Supplementary material for: Knowledge, attitudes, and practices related to TB among the general population of Ethiopia: Findings from a national cross-sectional survey
Source: PLoS One. 2019 Oct 28;14(10):e0224196. doi: 10.1371/journal.pone.0224196 (PMC6816561; doi:10.1371/journal.pone.0224196)
Supplement: S2 Table — (PDF) [file pone.0224196.s002.pdf]

Annex 1: Data collection tools

KNOWLEDGE, ATTITUDE, AND PRACTICE (KAP) RELATED TO TUBERCULOSIS AMONG THE GENERAL POPULATION, CURRENT TB PATIENTS, AND FAMILIES OF CURRENT TB PATIENTS IN ETHIOPIA

MODULE 1: QUESTIONNAIRE FOR GENERAL POPULATION SURVEY

SECTION 1: IDENTIFICATION

S.N	Question	Coding category
101	Region	Oromia..1<br>Amhara..2<br>SNNP..3<br>Tigrai..4<br>Benshangul Gumuz........................5<br>Gambella.......................................6<br>Addis Ababa..................................7<br>Dire Dawa......................................8<br>Harari..9
102	Zone	West Hararge............................... 11<br>North Shewa............................... 12<br>East Shewa ....13<br>West Guji ....14<br>North Shewa............................... 21<br>West Gojjam............................... 22<br>North Wollo................................ 23<br>Gondar City ....24<br>Sidama ....31<br>Segen area................................... 32<br>Sheka ....33<br>Siltie.. 34<br>Western Tigrai............................ 41<br>Eastern Tigrai..............................42<br>Kamashi.................................... ..51

		Agnuak ....61<br>Bole.. 71<br>Akaki Kality ....72<br>Gullelle ....73<br>Addis Ketema...............................74<br>Dire Dawa.................................... 81<br>Harari 91
103	Woreda	
104	Kebele name	
105	*Setting*	*Rural....................1*<br>*Urban..................2*
106	*House number*	
Location of the house		
107	Altitude (Meters)	
108	Latitude (degrees N)	
109	Longitude (degrees E)	

SECTION 2: INTERVIEWER VISITS

S.N	Question	Coding category
201	Date of visit (D/M/Y)	
202	Interviewer code	
203	Result of the visit	Completed...1<br>No competent respondent at home at time of visit...2<br>Refused...3
204	Supervisor code	

SECTION 3: SOCIO-DEMOGRAPHIC AND ECONOMIC CHARACTERISTICS

S.N	Questions and filters	Coding category (Fill)	Skip
401	Gender	Male…1<br>Female…2	
402	Age in completed	_________________Years	

	years		
403	Relationship to the head of the household	Head…1<br>Spouse…2<br>Son/Daughter…3<br>Other relative…4<br>Non-relative…5	
404	Religion	Orthodox Christian........................1<br>Muslim..2<br>Protestant......................................3<br>Catholic...4<br>Other (specify)............................96	
405	Marital Status	Married…1<br>Living together…2<br>Divorced/ Separated…3<br>Widowed…4<br>Never married/never lived together…5	
406	Educational status	Not able to read and write…1<br>Read and write only…2<br>Primary…3<br>Secondary…4<br>Above secondary…5	
407	Occupation	Employed…1<br>House wife…2<br>Farmer…3<br>Daily laborer…4<br>Trader…5<br>Student …6<br>No job/dependent…7<br>House maid…8<br>Other (Specify).............96	
408	Total number of household members		

409	What is the main source of drinking water for members of your household?	Piped water piped into the dwelling.................................1<br><br>Piped water piped to yard/plot...2<br><br>Bottled water.................... ....3<br><br>Piped water public tap/standpipe......................................4<br><br>Borehole...5<br><br>Protected dug well......... ....6<br><br>Unprotected dug well.......... ....7<br><br>Protected spring............... ....8<br><br>Unprotected spring........................... ....9<br><br>Rain water............. ....10<br><br>Tanker truck................. ....11<br><br>Cart with small tank......... ....12<br><br>Surface water.................... ....13<br><br>Other (specify)......................... 96	If 1,2, or 3, skip to Q411
410	How long does it take to go there, get water, and come back? (in minutes)		
411	What kind of toilet facility do members of your household usually use?	Flush to piped sewer system......................................1<br><br>Flush to septic tank . . . . .2<br><br>Flush to pit latrine . . . . .3<br><br>Flush to somewhere else . . .. .4<br><br>Flush, don't know where . . . . .5<br><br>Ventilated improved pit latrine (VIP). . . .. .6<br><br>Pit latrine with slab . . .. .7<br><br>Pit latrine without slab/open pit . . . . .8<br><br>No facility/bush/field . . . . .9<br><br>Other (specify)..96	If 9 or 96, skip to Q414
412	Do you share this toilet facility with other households?	Yes . . . . 1<br><br>No 0	If 0, skip to Q414
413	How many households use this toilet facility? (Write 98 if he/she doesn’t know the number of households)		
414	Does your household have electricity?	Yes..................... ....1<br><br>No..0	
415	Does your household have a	Yes.............. 1	

	wall clock?	No..0	
416	Does your household have a radio?	Yes..................... ....1<br><br>No................ 0	
417	Does your household have a television?	Yes...1<br><br>No.............. 0	
418	Does any member of your household have a mobile telephone?	Yes..1<br><br>No....... 0	
419	Does your household have a non-mobile telephone?	Yes...1<br><br>No............ 0	
420	Does your household have a refrigerator?	Yes.......... ....1<br><br>No......... 0	
421	Does your household have a table?	Yes...1<br><br>No......... 0	
422	Does your household have chairs?	Yes.............. ....1<br><br>No................. 0	
423	Does your household have a bed with cotton/sponge/spring mattress?	Yes.................. ....1<br><br>No.............. 0	
424	Does your household have an electric stove for baking enjera or bread (mitad)?	Yes.............. ....1<br><br>No................. 0	
425	Does your household have a kerosene lamp/pressure lamp?	Yes.................. ....1<br><br>No.............. 0	
426	What type of fuel does your household mainly use for cooking?	Electricity . . . .1<br><br>Liquefied Petroleum Gas (LPG) . . . . 2<br><br>Natural gas/biogas/kerosine.......... . . . . .3<br><br>Charcoal . . . .4<br><br>Wood . . . .5<br><br>Straw/shrubs/grass . . . .6<br><br>Animal dung 7	

		No food cooked in household . . .. 95<br><br>Other (specify)...96	
427	Is the cooking usually done in the house, in a separate building, or outdoors?	In the house . . . . 1<br><br>In a separate building/room..... . . . . 2<br><br>Outdoors . . . . 3<br><br>Other (specify)...96	
428	What is the main material of the floor?	Earth/sand . . . . 1<br><br>Dung . . . . 2<br><br>Unpolished wood...................... . . . . 3<br><br>Polished wood . . ..4<br><br>Cement . . . . 5<br><br>Ceramic tiles . . . . 6<br><br>Carpet . . .. 7<br><br>Other (specify)...96	
429	What is the main material of the roof?	No roof . . . . 1<br><br>Thatch/leaf/mud . . . . 2<br><br>Plastic sheets . . .. .3<br><br>Wood . . . .4<br><br>Corrugated iron /metal .... 5<br><br>Cement/concrete . . . . 6<br><br>Other (specify)..96	
430	What is the main material of the exterior walls?	No walls . . . . 1<br><br>Cane/bamboo . . . .2<br><br>Bamboo/wood with mud................... . . . . 3<br><br>Stone with mud . . . . 4<br><br>Uncovered adobe (earthen brick)......... . . . . 5<br><br>Cardboard . . . . 6<br><br>Cement . . . . 7<br><br>Stone with lime/cement . . . . .8<br><br>Bricks . . . . .9<br><br>Cement blocks 10	

		Covered adobe . . . . 11<br><br>Other (specify)............. 96	
431	How many rooms in this household are used for sleeping?		
432	Does any member of this household own a bicycle?	Yes............. ....1<br><br>No................ 0	
433	Does any member of this household own a motorcycle?	Yes................. ....1<br><br>No............. 0	
434	Does any member of this household own an animal-drawn cart?	Yes............. ....1<br><br>No................ 0	
435	Does any member of this household own a car or truck?	Yes................. ....1<br><br>No............. 0	
436	Does any member of this household own any agricultural land?	Yes............. ....1<br><br>No................ 0	If 0, skip to Q441
437	How many hectares of agricultural land do members of this household own?<br><br>1 hectare = 10,000 m^2 e.g 100 meter x 100 meter	Yes................. ....1<br><br>No............. 0	
438	What is the annual production of crops/ grains in quintal		
439	Does any member of this household own coffee plants?	Yes...1<br><br>No..0	If 0, skip to Q441
440	How many kilograms of coffee do you get on average each year?		
441	Does this household own any livestock, herds, other farm animals, or poultry?	Yes...1<br><br>No..0	If 0, skip to Q449
442	How many milk cows, oxen or bulls do this household own?<br><br>If none, enter '0';<br><br>If between 1-94, write the exact number;<br><br>If more than 95, enter '95';		

	If unknown, enter '98'.	
443	How many horses, donkeys, or mules do this household own? If none, enter '0';<br><br>If between 1-94, write the exact number;<br><br>If more than 95, enter '95';<br><br>If unknown, enter '98'.	
444	How many camels do this household own? If none, enter '0'; If between 1-94, write the exact number;<br><br>If more than 95, enter '95'; If unknown, enter '98'.	
445	How many goats do this household own? If none, enter '0'; If between 1-94, write the exact number;<br><br>If more than 95, enter '95'; If unknown, enter '98'.	
446	How many sheep do this household own? If none, enter '0'; If between 1-94, write the exact number;<br><br>If more than 95, enter '95'; If unknown, enter '98'.	
447	How many chickens do this household own? If none, enter '0'; If between 1-94, write the exact number;<br><br>If more than 95, enter '95'; If unknown, enter '98'.	
448	How many beehives do this household own? If none, enter '0'; If between 1-94, write the exact number;<br><br>If more than 95, enter '95'; If unknown, enter '98'.	
449	Does any member of this household have a bank or microfinance saving account?	Yes......1<br><br>No.........0

SECTION 4: KNOWLEDGE AND PERCEPTION ON TB

S.N	Questions and filters	Coding category (Fill)	Skip
501	Have you ever heard of an illness called TB?	Yes……...……………………1<br>No……………………………0	If no, End here
502	Where did you first learn/hear about TB?<br><br>Record all mentioned.	Newspapers and magazines….1<br>Radio….2<br>TV….3<br>Billboards…4<br>Brochures, posters and other printed materials…5<br>Health extension workers…6<br>Other health workers…7<br>Health development armies…8<br>Family, friends, neighbours and colleagues…9<br>Religious leaders…10<br>Teachers…11<br>Other (Specify)........96	
503	In your opinion, how serious a disease is TB?	Very serious…1<br>Somewhat serious…2<br>Not very serious…3	
504	How serious a problem do you think TB is in your community?	Very serious…1<br>Somewhat serious…2<br>Not very serious…3	
505	What is the cause of TB? (Don't read the options to him/her . Check all the options mention by him/her)<br><br>Probe: Anything else?	Germs….1<br>Evil eye….2<br>Satan/witchcraft….3<br>Other (Specify)...........96<br>Don't know…..99	
506	How can a person get TB? (Don't read the options to him/her . Check all the options mention by him/her)	Through the air when coughing or sneezing ….1<br>Through sharing utensils….2<br>Through touching a person with TB…...3	

	Probe: Any other?	Through food or water…..4<br><br>Through sexual contact with a person with TB….5<br><br>Through mosquito bites….6<br><br>Through drinking un-boiled milk….7<br><br>Exposure to cold….8<br><br>Other (Specify)............96<br><br>Don't know…..99	
507	In your opinion, who can be infected with TB? (Don't read the options to him/her . Check all the options mention by him/her)<br><br>Probe: Any other person?	Anybody…1<br><br>Only poor people…2<br><br>Only homeless people….3<br><br>Only alcoholics…4<br><br>Only drug users…5<br><br>Only people living with HIV/AIDS…6<br><br>Only people who have been in prison…7<br><br>Other (Specify)...............96	
508	What are the body parts affected with TB? (Don't read the options to him/her . Check all the options mention by him/her)<br><br>Probe: Any other part of the body?	Lung….1<br><br>Intestine….2<br><br>Bone….3<br><br>Lymph nodes....4<br><br>Other (Specify).......96<br><br>Don't know…..99	
509	What symptoms will a person with TB have? (Don't read the options to him/her . Check all the options mention by him/her)<br><br>Probe: Anything else?	Cough........1<br><br>Persistent cough (greater that two weeks)……..…2<br><br>Weight loss……...3<br><br>Poor appetite….4<br><br>Night sweating……...…5<br><br>Chest pain………..…..6<br><br>Fever…………………7<br><br>Blood in the sputum….7<br><br>Shortness of breath….9<br><br>Fatigue….10	

		Swelling..........11<br><br>Other (Specify)..........96<br><br>Don't know…..99	
510	Is TB preventable disease?	Yes…………………………………1<br>No…………..…………………….0<br><br>Don't know…..99	If 0 or 99, skip to Q512
511	What possible TB transmission prevention methods are there?<br><br>(Don't read the options to him/her . Check all the options mention by him/her)<br><br>Probe: Anything else?	Avoiding cough in front of people….1<br><br>Safe disposal of sputum….2<br><br>Ventilation of living room….3<br><br>Avoiding close contact with TB patients….4<br><br>Vaccination for children……5<br><br>Other (Specify)...........96<br><br>Don't know…..99	
512	Can TB be cured?	Yes…………………………1<br>No……………………….....0<br><br>Don't know…..99	If 0 or 99, skip to Q514
513	How can someone with TB be cured?	Herbal remedies…1<br><br>Home rest without medicine…2<br><br>Praying…3<br><br>Specific drugs given by health professionals…4<br><br>Other (Specify)........96<br><br>Don't know…..99	
514	Do you think that you are well informed about TB (on its prevention and control)?	Yes……………………………1<br>No………………….....……….0	
515	Do you wish to get more information about TB?	Yes…………………………………………1<br>No………………………………………….0	If 0, Skip to Q517
516	What are the sources of information that you think can most effectively reach people like you with information on TB? (Please choose the three most effective sources.)	Newspapers and magazines…1<br><br>Radio…2<br><br>TV…3<br><br>Billboards…4<br><br>Brochures, posters and other printed materials…5<br><br>Health extension workers…6<br><br>Other health workers…7	

		Health development armies…8<br><br>Family, friends, neighbours and colleagues…9<br><br>Religious leaders…10<br><br>Teachers…11<br><br>Other (Specify)...........96	
517	Have you ever heard about multidrug resistant TB?	Yes……………………………1<br>No…………………...……….0	If 0, Skip to Q601
518	What do you know about multidrug resistant TB?<br><br><br>Probe: Anything else?	It is created when TB patients do not take anti-TB drugs regularly………1<br><br>It is a dangerous form of TB…2<br><br>Can be transmitted to family and friends….3<br><br>Its treatment lasts for at least 2 years….4<br><br>Other (specify)__________________5<br><br>Can not explain multidrug resistant TB…6	

SECTION 5: ATTITUDE AND STIGMA RELATED QUESTIONS

S.N	Questions and filters	Coding category (Fill)	Skip
601	Do you think you can get TB?	Yes…………………………………………1<br>No………………………………………….0	
602	What would be your reaction if you were found out that you have TB?<br><br>(Check all that are mentioned.)	Cope with it….1<br><br>Fear…1<br><br>Surprise…2<br><br>Shame…3<br><br>Embarrassment…4<br><br>Sadness or hopelessness…5<br><br>Other (Specify)..........96	
603	Who would you talk to about your illness if you had TB?<br><br>(Check all that are mentioned.)	Doctor or other medical worker…1<br><br>Spouse…2<br><br>Parent…3<br><br>Children…4<br><br>Other family member…5<br><br>Close friend…6	

		No one…7<br><br>Other (Specify)........96	
604	What would you do if you thought you had symptoms of TB? (Check all that are mentioned)	Go to public health facility…1<br><br>Go to private health facility…2<br><br>Go to pharmacy…3<br><br>Go to spiritual/ traditional healer…4<br><br>Pursue other self-treatment options (herbs, etc.)…5<br><br>Other (Specify).............96<br><br>Don't know…..99	If 1,2, Skip to Q606
605	If you would not go to the health facility, what is the reason? (Check all that are mentioned)	Not sure where to go…1<br><br>Cost…2<br><br>Difficulties with transportation/distance to health facility…3<br><br>Do not trust health workers…4<br><br>Do not like attitude of health workers…5<br><br>Cannot leave work (overlapping work hours with health facility working hours)…6<br><br>Do not want to find out that something is really wrong…7<br><br>Other (Specify)..........96	
606	If you had symptoms of TB, at what point would you go to the health facility?	Immediately ………………………… 1<br><br>In few days…..2<br><br>One to two weeks….3<br><br>After two weeks….4<br><br>I will not go to health facility....5<br><br>Other (Specify)_____________________96	
607	How expensive do you think TB diagnosis and treatment is in this country?	It is free of charge…1<br><br>It is reasonably priced…2<br><br>It is somewhat/moderately expensive…3<br><br>It is very expensive…4<br><br>Don't know…..99	
608	Do you know people who have/had TB?	Yes…………………………………………1<br>No………………………………………….0	
609	Which statement is closest to your feeling about people with TB disease?	I feel compassion and desire to help.….1<br><br>I feel compassion but I tend to stay away from these people.…2	

	(Read the following choices and check one answer only)	It is their problem and I do not want to get TB by trying to help them.…3<br><br>I fear them because they may infect me.…4<br><br>I have no particular feeling.…5<br><br>Other (Specify)........96	
610	In your community, how is a person who has TB usually regarded/treated?	Most people reject him or her…1<br><br>Most people are friendly, but they generally try to avoid him or her…2<br><br>The community mostly supports and helps him or her…3<br><br>I don't have experience....4<br><br>Other (Specify)......96	
611	If yourself got tuberculosis or TB, you would want it to remain secret. (Read the options to the participant and inform him/her to select one option only)	Strongly disagree...........1<br><br>Disagree.........................2<br><br>Indifferent......................3<br><br>Agree.............................4<br><br>Strongly agree.................5	
612	If a member of your family got tuberculosis or TB, you would want it to remain secret. (Read the options to the participant and inform him/her to select one option only)	Strongly disagree...........1<br><br>Disagree.........................2<br><br>Indifferent......................3<br><br>Agree.............................4<br><br>Strongly agree.................5	
613	If you had TB, others would think less of you. (Read the options to the participant and inform him/her to select one option only)	Strongly disagree...........1<br><br>Disagree.........................2<br><br>Indifferent......................3<br><br>Agree.............................4<br><br>Strongly agree.................5	
614	If you had TB, you would be ashamed or embarrassed. (Read the options to the participant and inform him/her to select one option only)	Strongly disagree...........1<br><br>Disagree.........................2<br><br>Indifferent......................3<br><br>Agree.............................4<br><br>Strongly agree.................5	
615	If you had TB, others would avoid you. (Read the options to the participant and inform him/her to	Strongly disagree...........1	

	select one option only)	Disagree.........................2<br>Indifferent......................3<br>Agree.............................4<br>Strongly agree.................5	
616	*Do you have partner?*	*Yes………………………………………1*<br>*No……………………………………….0*	*If 1, skip to Q618*
617	*Only for the respondent who has no partner -If you had TB, you would have a problem of finding a partner for marriage even after cure. ((Read the options to the participant and inform him/her to select one option only)*	Strongly disagree...........1<br>Disagree.........................2<br>Indifferent......................3<br>Agree.............................4<br>Strongly agree.................5	
618	*Only for the respondent who has partner- If you had TB, your partner would refuse to have sex with you. ((Read the options to the participant and inform him/her to select one option only)*	Strongly disagree...........1<br>Disagree.........................2<br>Indifferent......................3<br>Agree.............................4<br>Strongly agree.................5	*Those who responded 0 to Q616 shall not respond to this question*
619	If you had TB, you would be asked to stay away from a social group. (Read the options to the participant and inform him/her to select one option only)	Strongly disagree...........1<br>Disagree.........................2<br>Indifferent......................3<br>Agree.............................4<br>Strongly agree.................5	
620	If you had TB, you would not disclose even to a confidant (Somebody to who secrets are usually told). ((Read the options to the participant and inform him/her to select one option only)	Strongly disagree...........1<br>Disagree.........................2<br>Indifferent......................3<br>Agree.............................4<br>Strongly agree.................5	
621	If you had TB, you would think less of yourself. (Read the options to the participant and inform him/her to select one option only)	Strongly disagree...........1<br>Disagree.........................2<br>Indifferent......................3<br>Agree.............................4<br>Strongly agree.................5	
622	If you had TB, others would think less of your family. (Read the	Strongly disagree...........1	

	options to the participant and inform him/her to select one option only)	Disagree.........................2<br><br>Indifferent......................3<br><br>Agree.............................4<br><br>Strongly agree.................5	
623	Do you have children?	Yes....1<br><br>No....0	If ‘0’ skip to 701
624	If you had TB, it would be a problem for your children. (Read the options to the participant and inform him/her to select one option only)	Strongly disagree...........1<br><br>Disagree.........................2<br><br>Indifferent......................3<br><br>Agree.............................4<br><br>Strongly agree.................5	

SECTION 6: PRACTICE RELATED QUESTIONS

S.N	Questions and filters	Coding category (Fill)	Skip
701	Have you ever been diagnosed with TB?	Yes……………….1<br>No………………..0	If 0, Skip to Q707
702	When have you been diagnosed with TB? (Month/Year- Ethiopian Calendar)		
703	Where was your first point of care at that time?	Sought care from health institutions….1<br>Sought care from pharmacies…2<br>Contacted the HDA to advise me to seek care from the health institutions….3<br>Contacted the HEW to advise me to seek care from health institutions…..4<br>Visited spiritual/ traditional healers…5<br>I did nothing….6<br>Other (specify)__________96	
704	Where were you first diagnosed with TB?	Health center…1<br>*Public hospital…2*<br>*Private hospital.....3*<br>*Private clinic.....4*<br>Other (specify)......96	
705	Did you complete the treatment?	Yes……………….1<br>No………………..0	
706	How satisfied were you with the health service provided?	Very satisfied….1<br>Somehow satisfied….2<br>Indifferent….3<br>Somehow dissatisfied…4<br>Very dissatisfied….5	
707	In the past one year, have you ever experienced cough for 2 weeks or more?	Yes……………….1<br>No………………..0	If 0, Skip to Q709
708	What did you do when you had cough for 2 weeks or more? (more than one answer possible)	Sought care from health institutions….1<br>Sought care from pharmacies…2<br>Contacted the HDA to advise me to seek care from	

		the health institutions….3<br><br>Contacted the HEW to advise me to seek care from health institutions…..4<br><br>Visited spiritual/ traditional healers…5<br><br>I did nothing….6<br><br>Other (specify)………..…96	
709	In the past one year, have you ever encountered a person who has cough for 2 weeks or more in your community?	Yes……………….1<br><br>No………………..0	If 0, skip to Q711
710	What did you do when you encountered a person who has cough for 2 weeks or more in your community? (more than one answer possible)	I advised him/her to seek care from public health institutions….1<br><br>I advised him/her to seek care from private health facilities…2<br><br>I advised him/her to seek care from pharmacies…3<br><br>I informed the HDA to advise him/her to seek care from the health institutions….4<br><br>I informed the HEW to advise him/her to seek care from health institutions…..5<br><br>I advised him/her to seek care from spiritual/ traditional healers…6<br><br>I did nothing….7<br><br>Other (specify).....96	
711	Were you involved in the past on identification of TB cases?	Yes……………………….1<br><br>No…………………………0	If 0, skip to Q713
712	What did you do?	Referred family member to health facility…1<br><br>Referred community member to health facility…2<br><br>Involved in TB screening at community level…3<br><br>Other (Specify)......96	
713	Were you involved in the past in tracing of TB treatment defaulters?	Yes……………………….1<br><br>No…………………………0	
714	Have you advised parents of children under-one year to have their infants vaccinated for TB?	Yes……………………….1<br><br>No…………………………0	
715	Were there circumstances when you advised TB patients to take their drugs properly based on the advice of health professionals?	Yes……………………….1<br><br>No…………………………0	
716	Have you ever acted as TB treatment supporter for a TB patient in observing TB patients taking	Yes……………………….1	

	oral treatment?	No…………………………0	
717	In the past one year, *was* there any family member who has cough for 2 or more weeks?	Yes………………………1<br><br>No…………………………0	If 0, Skip to Q719
718	What did you do when your family member had cough for 2 or more weeks?	We did nothing……………1<br><br>We took him/her to public health institutions for investigation…2<br><br>We took him/her to private health institution for investigation…3<br><br>We gave him/her medicine without consulting health professionals…4<br><br>We took him/her to Pharmacy…5<br><br>We took him/her to Spiritual/ Traditional healer…6<br><br>Other (specify).......96	
719	Have you ever been involved in a community activity engaged in awareness creation, TB prevention and TB case finding efforts?	Yes………………………1<br><br>No…………………………0	If 0, End here.
720	Who organized the community activity?	*Health Development Army (HDA).....1*<br><br>*HEW/Health post.....2*<br><br>*Kebele…3*<br><br>*Health center/hospital staff…4*<br><br>*Woreda health office....5*<br><br>*NGO…6*<br><br>*Community organization like Idir…7*<br><br>*Other (Specify).......96*	

End of questions

Thank you very much for your cooperation

MODULE 3: QUESTIONNAIRE FOR KAP SURVEY – FAMILIES OF TB PATIENTS

SECTION 1: IDENTIFICATION

S.N	Question	Coding category
101	Region	Oromia..1<br>Amhara...2<br>SNNP..3<br>Tigrai..4<br>Benshangul Gumuz.......................5<br>Gambella......................................6<br>Addis Ababa.................................7<br>Dire Dawa.....................................8<br>Harari..9
102	Zone	West Hararge............................... 11<br>North Shewa............................... 12<br>East Shewa ....13<br>West Guji ....14<br>North Shewa............................... 21<br>West Gojjam............................... 22<br>North Wollo................................ 23<br>Gondar City ....24<br>Sidama ....31<br>Segen area.................................. 32<br>Sheka ....33<br>Siltie.. 34<br>Western Tigrai............................ 41<br>Eastern Tigrai..............................42<br>Kamashi.................................. ..51<br>Agnuak ....61<br>Bole... 71<br>Akaki Kality ....72<br>Gullelle 73

		Addis Ketema...............................74<br>Dire Dawa.................................... 81<br>Harari 91
103	Woreda	
104	*Health center name*	
105	*Name of kebele where the health center is located*	
106	*Setting of the kebele where the health center is located*	*Rural....................1*<br>*Urban..................2*
107	*Name of the kebele where the family member of TB patient comes from*	
108	*Setting of the kebele where the family member of TB patient comes from*	*Rural....................1*<br>*Urban..................2*
Location of the health center		
109	Altitude (Meters)	
110	Latitude (degrees N)	
111	Longitude (degrees E)	

SECTION 2: INTERVIEWER VISITS

S.N	Question	Coding category
201	Date of interview (D/M/Y)	
202	Interviewer code	
203	Result of the interview	Completed...1<br>Refused...2
204	Supervisor code	

SECTION 3:SOCIO-DEMOGRAPHIC AND ECONOMIC CHARACTERISTICS

S.N	Questions and filters	Coding category (Fill)	Skip

401	Gender	Male…1<br><br>Female…2	
402	Age in completed years	_________________Years	
403	Relationship to the head of the household	Head…1<br><br>Spouse…2<br><br>Son/Daughter…3<br><br>Other relative…4<br><br>Non-relative…5	
404	Religion	Orthodox Christian........................1<br><br>Muslim..2<br><br>Protestant......................................3<br><br>Catholic..4<br><br>Other (specify)............................96	
405	Marital Status	Married…1<br><br>Living together…2<br><br>Divorced/ Separated…3<br><br>Widowed…4<br><br>Never married/never lived together…5	
406	Educational status	Not able to read and write…1<br><br>Read and write only…2<br><br>Primary…3<br><br>Secondary…4<br><br>Above secondary…5	
407	Occupation	Employed…1<br><br>House wife…2<br><br>Farmer…3<br><br>Daily laborer…4<br><br>Trader…5<br><br>Student …6<br><br>No job/dependent…7<br><br>House maid…8	

		Other (Specify).............96	
408	Total number of household members		
409	Total number of children under-one year old in the family		
410	Total number of children under-five years in the family		
411	Total number of people age 5 years to less than 18 years in the family		
412	Total number of adults (18 years and above) in the family		
413	What is the main source of drinking water for members of your household?	Piped water piped into the dwelling.....1<br><br>Piped water piped to yard/plot......2<br><br>Bottled water................................3<br><br>Piped water public tap/standpipe..........4<br><br>Borehole......................................5<br><br>Protected dug well..........................6<br><br>Unprotected dug well......................7<br><br>Protected spring...............................8<br><br>Unprotected spring..........................9<br><br>Rain water............. ....10<br><br>Tanker truck................ ....11<br><br>Cart with small tank............... ....12<br><br>Surface water................................13<br><br>Other (specify)............................. 96	If 1,2, or 3, skip to Q415
414	How long does it take to go there, get water, and come back? (in minutes)		
415	What kind of toilet facility do members of your household usually use?	Flush to piped sewer system...........1<br><br>Flush to septic tank . . . . .2<br><br>Flush to pit latrine .... .3<br><br>Flush to somewhere else . . .. .4<br><br>Flush, don't know where . .. . .5<br><br>Ventilated improved pit latrine (VIP). . .6<br><br>Pit latrine with slab . . .. .7<br><br>Pit latrine without slab/open pit . . . 8<br><br>No facility/bush/field . . . . .9<br><br>Other (specify)............................96	If 9 or 96, skip to Q418

416	Do you share this toilet facility with other households?	Yes . . . . 1<br><br>No 0	If 0, skip to Q418
417	How many households use this toilet facility? (Write 98 if he/she doesn't know the number of households)		
418	Does your household have electricity?	Yes..................................1<br><br>No...................................0	
419	Does your household have a wall clock?	Yes..................................1<br><br>No...................................0	
420	Does your household have a radio?	Yes..................................1<br><br>No...................................0	
421	Does your household have a television?	Yes..................................1<br><br>No...................................0	
422	Does any member of your household have a mobile telephone?	Yes..................................1<br><br>No...................................0	
423	Does your household have a non-mobile telephone?	Yes..................................1<br><br>No...................................0	
424	Does your household have a refrigerator?	Yes..................................1<br><br>No...................................0	
425	Does your household have a table?	Yes..................................1<br><br>No...................................0	
426	Does your household have chairs?	Yes..................................1<br><br>No...................................0	
427	Does your household have a bed with cotton/sponge/spring mattress?	Yes..................................1<br><br>No...................................0	
428	Does your household have an electric stove for baking enjera or bread (mitad)?	Yes..................................1<br><br>No...................................0	
429	Does your household have a kerosene lamp/pressure lamp?	Yes..................................1<br><br>No...................................0	
430	What type of fuel does your household mainly use for cooking?	Electricity . . . .1<br><br>Liquefied Petroleum Gas (LPG) . . . . 2<br><br>Natural gas/biogas/kerosine.......... 3	

		Charcoal . . . .4<br>Wood . . . .5<br>Straw/shrubs/grass . . . .6<br>Animal dung . . . . 7<br>No food cooked in household . . .. 95<br>Other (specify)...96	
431	Is the cooking usually done in the house, in a separate building, or outdoors?	In the house . . . . 1<br>In a separate building/room..... . . . . 2<br>Outdoors . . . . 3<br>Other (specify)...96	
432	What is the main material of the floor?	Earth/sand . . . . 1<br>Dung . . . . 2<br>Unpolished wood...................... . . . . 3<br>Polished wood . . ..4<br>Cement . . . . 5<br>Ceramic tiles . . . . 6<br>Carpet . . .. 7<br>Other (specify).......................................96	
433	What is the main material of the roof?	No roof . . . . 1<br>Thatch/leaf/mud . . . . 2<br>Plastic sheets . . .. .3<br>Wood . . . .4<br>Corrugated iron /metal .... 5<br>Cement/concrete . . . . 6<br>Other (specify)................................96	
434	What is the main material of the exterior walls?	No walls . . . . 1<br>Cane/bamboo ....2<br>Bamboo/wood with mud....... . 3<br>Stone with mud . . . . 4<br>Uncovered adobe (earthen brick)..... 5<br>Cardboard 6	

		Cement . . . . 7<br><br>Stone with lime/cement . . . . . .8<br><br>Bricks . . . . .9<br><br>Cement blocks . . . . .10<br><br>Covered adobe . . . . 11<br><br>Other (specify).................96	
435	How many rooms in your household are used for sleeping?		
436	Does any member of your household own a bicycle?	Yes...................................1<br><br>No....................................0	
437	Does any member of your household own a motorcycle?	Yes...................................1<br><br>No....................................0	
438	Does any member of your household own an animal-drawn cart?	Yes...................................1<br><br>No....................................0	
439	Does any member of your household own a car or truck?	Yes...................................1<br><br>No....................................0	
440	Does any member of your household own any agricultural land?	Yes...................................1<br><br>No....................................0	If 0, skip to Q445
441	How many hectares of agricultural land do members of your household own?<br><br>1 hectare=10,000 m^2 e.g 100 meter x 100 meter	Yes...................................1<br><br>No....................................0	
442	What is the annual production of crops/ grains in quintal		
443	Does any member of your household own coffee plants?	Yes...........................1<br><br>No...........................0	If 0, skip to Q445
444	How many kilograms of coffee do you get on average each year?		
445	Does your household own any livestock, herds, other farm animals, or poultry?	Yes.........................1<br><br>No..........................0	If 0, skip to Q453

446	How many milk cows, oxen or bulls do your household own?<br><br>If none, enter '0'; If between 1-94, write the exact number; If more than 95, enter '95'; If unknown, enter '98'.		
447	How many horses, donkeys, or mules do your household own?<br><br>If none, enter '0'; If between 1-94, write the exact number; If more than 95, enter '95'; If unknown, enter '98'.		
448	How many camels do your household own?<br><br>If none, enter '0'; If between 1-94, write the exact number; If more than 95, enter '95'; If unknown, enter '98'.		
449	How many goats do your household own?<br><br>If none, enter '0'; If between 1-94, write the exact number; If more than 95, enter '95'; If unknown, enter '98'.		
450	How many sheep do your household own?<br><br>If none, enter '0'; If between 1-94, write the exact number; If more than 95, enter '95'; If unknown, enter '98'.		
451	How many chickens do your household own?<br><br>If none, enter '0'; If between 1-94, write the exact number; If more than 95, enter '95'; If unknown, enter '98'.		
452	How many beehives do your household own?<br><br>If none, enter '0'; If between 1-94, write the exact number; If more than 95, enter '95'; If unknown, enter '98'.		
453	Does any member of your household have a bank or microfinance saving account?	Yes........................1<br><br>No......... 0	

SECTION 4: KNOWLEDGE AND PERCEPTION ON TB

S.N	Questions and filters	Coding category (Fill)	Skip
501	Have you ever heard of an illness called TB?	Yes……...……………………1<br><br>No…………………………….0	If no, End here
502	Where did you first learn/hear about TB?<br><br>Record all mentioned.	Newspapers and magazines….1<br><br>Radio….2<br><br>TV….3<br><br>Billboards…4	

		Brochures, posters and other printed materials…5<br>Health extension workers…6<br>Other health workers…7<br>Health development armies…8<br>Family, friends, neighbours and colleagues…9<br>Religious leaders…10<br>Teachers…11<br>Other (Specify)........96	
503	In your opinion, how serious a disease is TB?	Very serious…1<br>Somewhat serious…2<br>Not very serious…3	
504	How serious a problem do you think TB is in your community?	Very serious…1<br>Somewhat serious…2<br>Not very serious…3	
505	What is the cause of TB? (Don't read the options to him/her. Check all the options mention by him/her)<br><br>Probe: Anything else?	Germs….1<br>Evil eye….2<br>Satan/witchcraft….3<br>Other (Specify)...........96<br>Don't know…..99	
506	How can a person get TB? (Don't read the options to him/her . Check all the options mention by him/her)<br><br>Probe: Any other?	Through the air when coughing or sneezing ….1<br>Through sharing utensils….2<br>Through touching a person with TB…...3<br>Through food or water…..4<br>Through sexual contact with a person with TB….5<br>Through mosquito bites….6<br>Through drinking un-boiled milk….7<br>Exposure to cold….8<br>Other (Specify)............96	

		Don't know…..99	
507	In your opinion, who can be infected with TB? (Don't read the options to him/her . Check all the options mention by him/her)<br><br>Probe: Any other person?	Anybody…1<br>Only poor people…2<br>Only homeless people….3<br>Only alcoholics…4<br>Only drug users…5<br>Only people living with HIV/AIDS…6<br>Only people who have been in prison…7<br>Other (Specify)..............96	
508	What are the body parts affected with TB? (Don't read the options to him/her . Check all the options mention by him/her)<br><br>Probe: Any other part of the body?	Lung….1<br>Intestine….2<br>Bone….3<br>Lymph nodes....4<br>Other (Specify).......96<br>Don't know…..99	
509	What symptoms will a person with TB have? (Don't read the options to him/her . Check all the options mention by him/her)<br><br>Probe: Anything else?	Cough.......1<br>Persistent cough (greater that two weeks)……..…2<br>Weight loss……...3<br>Poor appetite….4<br>Night sweating……...…5<br>Chest pain………..…..6<br>Fever…………………7<br>Blood in the sputum….8<br>Shortness of breath….9<br>Fatigue….10<br>*Swelling...........11*<br>Other (Specify)..........96<br>Don't know…..99	
510	Is TB preventable disease?	Yes…………………………………1	If 0 or 99,

		No…………...…………………….0<br><br>Don't know…..99	skip to Q512
511	What possible TB transmission prevention methods are there?<br><br>(Don't read the options to him/her . Check all the options mention by him/her)<br><br>Probe: Anything else?	Avoiding cough in front of people….1<br><br>Safe disposal of sputum….2<br><br>Ventilation of living room….3<br><br>Avoiding close contact with TB patients….4<br><br>Vaccination for children……5<br><br>Other (Specify)...........96<br><br>Don't know…..99	
512	Can TB be cured?	Yes…………………………1<br>No………………………....0<br><br>Don't know…..99	If 0 or 99, skip to Q514
513	How can someone with TB be cured?	Herbal remedies…1<br><br>Home rest without medicine…2<br><br>Praying…3<br><br>Specific drugs given by health professionals…4<br><br>Other (Specify)........96<br><br>Don't know…..99	
514	Do you think that you are well informed about TB (on its prevention and control)?	Yes……………………………1<br>No………………....……….0	
515	Do you wish to get more information about TB?	Yes…………………………………………1<br>No……………………………………….0	If 0, Skip to Q601
516	What are the sources of information that you think can most effectively reach people like you with information on TB? (Please choose the three most effective sources.)	Newspapers and magazines…1<br><br>Radio…2<br><br>TV…3<br><br>Billboards…4<br><br>Brochures, posters and other printed materials…5<br><br>Health extension workers…6<br><br>Other health workers…7<br><br>Health development armies…8<br><br>Family, friends, neighbours and colleagues…9	

		Religious leaders…10<br><br>Teachers…11<br><br>Other (Specify)...........96	
517	Have you ever heard about multidrug resistant TB?	Yes……………………………1<br>No………………….....……….0	If 0, Skip to Q601
518	What do you know about multidrug resistant TB?<br><br>Probe: Anything else?	It is created when TB patients do not take anti-TB drugs regularly………1<br><br>It is a dangerous form of TB…2<br><br>Can be transmitted to family and friends….3<br><br>Its treatment lasts for at least 2 years….4<br><br>Other (specify)__________________5<br><br>Can not explain multidrug resistant TB…6	

SECTION 5: ATTITUDE AND STIGMA RELATED QUESTIONS

S.N	Questions and filters	Coding category (Fill)	Skip
601	Do you think you can get TB?	Yes…………………………………………1<br>No………………………………………….0	
602	What would be your reaction if you were found out that you have TB?<br><br>(Check all that are mentioned.)	Cope with it….1<br><br>Fear…1<br><br>Surprise…2<br><br>Shame…3<br><br>Embarrassment…4<br><br>Sadness or hopelessness…5<br><br>Other (Specify)..........96	
603	Who would you talk to about your illness if you had TB?<br><br>(Check all that are mentioned.)	Doctor or other medical worker…1<br><br>Spouse…2<br><br>Parent…3<br><br>Children…4<br><br>Other family member…5<br><br>Close friend…6<br><br>No one…7<br><br>Other (Specify)........96	

604	What would you do if you thought you had symptoms of TB? (Check all that are mentioned)	Go to public health facility…1<br><br>Go to private health facility…2<br><br>Go to pharmacy…3<br><br>Go to spiritual/ traditional healer…4<br><br>Pursue other self-treatment options (herbs, etc.)…5<br><br>Other (Specify).............96<br><br>Don't know…..99	If 1,2, Skip to Q606
605	If you would not go to the health facility, what is the reason? (Check all that are mentioned)	Not sure where to go…1<br><br>Cost…2<br><br>Difficulties with transportation/distance to health facility…3<br><br>Do not trust health workers…4<br><br>Do not like attitude of health workers…5<br><br>Cannot leave work (overlapping work hours with health facility working hours)…6<br><br>Do not want to find out that something is really wrong…7<br><br>Other (Specify)..........96	
606	If you had symptoms of TB, at what point would you go to the health facility?	Immediately ………………………… 1<br><br>In few days…..2<br><br>One to two weeks….3<br><br>After two weeks….4<br><br>I will not go to health facility....5<br><br>Other (Specify)_____________________96	
607	How expensive do you think TB diagnosis and treatment is in this country?	It is free of charge…1<br><br>It is reasonably priced…2<br><br>It is somewhat/moderately expensive…3<br><br>It is very expensive…4<br><br>Don't know…..99	
608	Do you know people who have/had TB?	Yes…………………………………………1<br>No…………………………………………0	
609	Which statement is closest to your feeling about people with TB disease?<br><br>(Read the following choices and check one answer only)	I feel compassion and desire to help…..1<br><br>I feel compassion but I tend to stay away from these people.…2<br><br>It is their problem and I cannot get TB by trying to help them.…3	

		I fear them because they may infect me.…4<br><br>I have no particular feeling.…5<br><br>Other (Specify)........96	
610	In your community, how is a person who has TB usually regarded/treated?	Most people reject him or her…1<br><br>Most people are friendly, but they generally try to avoid him or her…2<br><br>The community mostly supports and helps him or her…3<br><br>Other (Specify)......96	
611	The fact that your family member has TB should be kept secret from your neighbours/community. (Read the options to the participant and inform him/her to select one option only)	Strongly disagree...........1<br><br>Disagree.........................2<br><br>Indifferent......................3<br><br>Agree.............................4<br><br>Strongly agree.................5	
612	If you yourself got tuberculosis or TB, you would want it to remain secret. (Read the options to the participant and inform him/her to select one option only)	Strongly disagree...........1<br><br>Disagree.........................2<br><br>Indifferent......................3<br><br>Agree.............................4<br><br>Strongly agree.................5	
613	It is shameful to have a family member with TB. (Read the options to the participant and inform him/her to select one option only)	Strongly disagree...........1<br><br>Disagree.........................2<br><br>Indifferent......................3<br><br>Agree.............................4<br><br>Strongly agree.................5	
614	If you had TB, others would think less of you. (Read the options to the participant and inform him/her to select one option only)	Strongly disagree...........1<br><br>Disagree.........................2<br><br>Indifferent......................3<br><br>Agree.............................4<br><br>Strongly agree.................5	
615	If you had TB, you would be ashamed/embarrassed. (Read the options to the participant and inform him/her to select one option only)	Strongly disagree...........1<br><br>Disagree.........................2<br><br>Indifferent......................3<br><br>Agree.............................4	

		Strongly agree................5	
616	If you had TB, others would avoid you. (Read the options to the participant and inform him/her to select one option only)	Strongly disagree...........1<br>Disagree.........................2<br>Indifferent......................3<br>Agree.............................4<br>Strongly agree................5	
617	*Do you have partner?*	*Yes……………………………………1*<br>*No…………………………………….0*	*If 1, skip to Q619*
618	*Only for the respondent who has no partner -If you had TB, you would have a problem of finding a partner for marriage even after cure. ((Read the options to the participant and inform him/her to select one option only)*	Strongly disagree...........1<br>Disagree.........................2<br>Indifferent......................3<br>Agree.............................4<br>Strongly agree................5	
619	*Only for the respondent who has partner- If you had TB, your partner would refuse to have sex with you. ((Read the options to the participant and inform him/her to select one option only)*	Strongly disagree...........1<br>Disagree.........................2<br>Indifferent......................3<br>Agree.............................4<br>Strongly agree................5	*Those who responded 0 to Q617 shall not respond to this question*
620	If you had TB, you would be asked to stay away from a social group. (Read the options to the participant and inform him/her to select one option only)	Strongly disagree...........1<br>Disagree.........................2<br>Indifferent......................3<br>Agree.............................4<br>Strongly agree................5	
621	If you had TB, you would not disclose even to a confidant. (Read the options to the participant and inform him/her to select one option only)	Strongly disagree...........1<br>Disagree.........................2<br>Indifferent......................3<br>Agree.............................4<br>Strongly agree................5	
622	If you had TB, you would think less of yourself. (Read the options to the participant and inform him/her to select one option only)	Strongly disagree...........1<br>Disagree.........................2<br>Indifferent......................3<br>Agree.............................4	

		Strongly agree................5	
623	If you had TB, others would think less of your family. (Read the options to the participant and inform him/her to select one option only)	Strongly disagree...........1<br><br>Disagree.........................2<br><br>Indifferent......................3<br><br>Agree.............................4<br><br>Strongly agree................5	
624	Do you have children	Yes....1<br><br>No....0	If '0' skip to 701
625	If you had TB, it would be a problem for your children. (Read the options to the participant and inform him/her to select one option only)	Strongly disagree...........1<br><br>Disagree.........................2<br><br>Indifferent......................3<br><br>Agree.............................4<br><br>Strongly agree................5	

SECTION 6: PRACTICE RELATED QUESTIONS

S.N	Questions and filters	Coding category (Fill)	Skip
701	Do you live with the family member who has TB?	Yes…………………1<br>No…......…………0	
702	Have you received any counselling or information from health professionals about the prevention and control of TB after your family member is diagnosed to have TB?	Yes…………………1<br>No…......…………0	
703	Are you supporting your family member who has TB to take the TB drugs without interruption?	Yes…………………1<br>No…......…………0	If 0, Skip to Q705
704	How do you support your family member who has TB in taking the drugs without interruption? (More than one answer possible)	I advise him/her to take the drug without interruption…1<br><br>I accompany him/her when he/she goes to health institutions to collect/take the drugs…2<br><br>I observe him/her when he/she take the drug….3<br><br>I facilitate transportation when going to collect/take the drugs….4<br><br>I provide financial support to him/her..5<br><br>Other (specify)…96	
705	Have you informed any family member that	Yes…………………1	If 0, Skip to

	your family member has TB?	No…........…………0	Q707
706	Among the family members, who did you inform that your family member has TB? (More than one answer possible)	Husband…..………………………1<br><br>Wife………………………………..2<br><br>Sister……………………….……….3<br><br>Brother………………..……….4<br><br>Son……..…………………………5<br><br>Daughter.......................................6<br><br>Other (specify)……………..96	
707	Have you informed anyone who is not family member that your family member has TB?	Yes…………………1<br>No…........…………0	If 0, Skip to Q709
708	Among people who are not family members, who did you inform that your family member has TB?	Religious leaders…………..1<br><br>Neighbors…………..2<br><br>Colleagues at work place……..3<br><br>Health professional who are not treating the family member who has TB…….4<br><br>Other (specify)…..96	
709	Have you informed the health extension worker in your kebele that your family member has TB?	Yes…………………1<br>No…........…………0	
710	Do you support or encourage your family member who has TB to eat more and a variety of food?	Yes…………………1<br>No…........…………0	
711	How do you support him/her to eat more and have a variety of food? (More than one answer possible)	I give advice………..1<br><br>I bring a variety of food from the market…2<br><br>I provide financial support….3<br><br>I cook the food and serve him/her….4<br><br>Other (specify)….96	
712	Do you open windows/doors in your house after you knew that your family member has TB?	Yes…………………1<br>No…........…………0	
713	Are you investigated for TB?	Yes…………………1<br>No…........…………0	
714	Have you informed other family members to go to the health institutions for investigation?	Yes…………………1<br>No…........…………0	If 0, Skip to Q716

715	Did they go for investigation?	Yes…………………1<br>No…........………….0	
716	Are there children whose age is under five years in your family?	Yes…………………1<br>No…........………….0	If 0, Skip to Q718
717	Did they get preventive therapy for TB?	Yes…………………1<br>No…........………….0	
718	Is there a child whose age is under one year in the family?	Yes…………………1<br>No…........………….0	If 0, Skip to Q720
719	Is he/she vaccinated for TB?	Yes…………………1<br>No…........………….0	
720	Are there family members in the house who have HIV?	Yes…………….2<br>No……………1<br>I don't know…………0	If 1 & 0, Skip to Q723
721	Did you advise them to get preventive therapy for TB?	Yes…………………1<br>No…........………….0	
722	Did they get preventive therapy for TB?	Yes…………………1<br>No…........………….0	
723	Have you advised family members how they should prevent TB?	Yes…………………1<br>No…........………….0	
724	Do you teach the community on the prevention and control of tuberculosis	Yes…………………1<br>No…........………….0	

End of questions

Thank you very much for your cooperation

MODULE 2: QUESTIONNAIRE FOR KAP SURVEY – TB PATIENTS

SECTION 1: IDENTIFICATION

S.N	Question	Coding category
101	Region	Oromia...1<br>Amhara..2<br>SNNP..3<br>Tigrai..4<br>Benshangul Gumuz........................5<br>Gambella.......................................6<br>Addis Ababa..................................7<br>Dire Dawa......................................8<br>Harari...9
102	Zone	West Hararge................................ 11<br>North Shewa................................ 12<br>East Shewa ....13<br>West Guji ....14<br>North Shewa................................ 21<br>West Gojjam................................ 22<br>North Wollo................................. 23<br>Gondar City ....24<br>Sidama ....31<br>Segen area.................................... 32<br>Sheka ....33<br>Siltie... 34<br>Western Tigrai............................. 41<br>Eastern Tigrai...............................42<br>Kamashi..................................... ..51<br>Agnuak ....61<br>Bole.. 71<br>Akaki Kality 72

		Gullelle ....73<br>Addis Ketema...............................74<br>Dire Dawa.................................... 81<br>Harari 91
103	Woreda	
104	*Health center name*	
105	*Name of kebele where the health center is located*	
106	*Setting of the kebele where the health center is located*	*Rural....................1*<br>*Urban...................2*
107	*Name of kebele where the patient comes from*	
108	*Setting of the kebele where the patient comes from*	*Rural....................1*<br>*Urban...................2*
Location of the health center		
109	Altitude (Meters)	
110	Latitude (degrees N)	
111	Longitude (degrees E)	

SECTION 2: INTERVIEWER VISITS

S.N	Question	Coding category
201	Date of visit (D/M/Y)	
202	Interviewer code	
203	Result of the visit	Completed..1<br>Refused..2
204	Supervisor code	

SECTION 3: SOCIO-DEMOGRAPHIC AND ECONOMIC CHARACTERISTICS

S.N	Questions and filters	Coding category (Fill)	Skip
401	Gender	Male…1	

		Female…2	
402	Age in completed years	_________________Years	
403	Relationship to the head of the household	Head…1<br><br>Spouse…2<br><br>Son/Daughter…3<br><br>Other relative…4<br><br>Non-relative…5	
404	Religion	Orthodox Christian........................1<br><br>Muslim..2<br><br>Protestant.....................................3<br><br>Catholic..4<br><br>Other (specify)............................96	
405	Marital Status	Married…1<br><br>Living together…2<br><br>Divorced/ Separated…3<br><br>Widowed…4<br><br>Never married/never lived together…5	
406	Educational status	Not able to read and write…1<br><br>Read and write only…2<br><br>Primary…3<br><br>Secondary…4<br><br>Above secondary…5	
407	Occupation	Employed…1<br><br>House wife…2<br><br>Farmer…3<br><br>Daily laborer…4<br><br>Trader…5<br><br>Student …6<br><br>No job/dependent…7<br><br>House maid…8<br><br>Other (Specify).............96	

408	Total number of household members		
409	Total number of children under-one year old in the family		
410	Total number of children under-five years in the family		
411	Total number of people age 5 years to less than 18 years in the family		
412	Total number of adults (18 years and above) in the family		
413	What is the main source of drinking water for members of your household?	Piped water piped into the dwelling.....1<br>Piped water piped to yard/plot......2<br>Bottled water................................3<br>Piped water public tap/standpipe..........4<br>Borehole......................................5<br>Protected dug well..........................6<br>Unprotected dug well......................7<br>Protected spring...............................8<br>Unprotected spring..........................9<br>Rain water............ ....10<br>Tanker truck................ ....11<br>Cart with small tank.............. ....12<br>Surface water.................................13<br>Other (specify)............................ 96	If 1,2, or 3, skip to Q415
414	How long does it take to go there, get water, and come back? (in minutes)		
415	What kind of toilet facility do members of your household usually use?	Flush to piped sewer system...........1<br>Flush to septic tank . . . . .2<br>Flush to pit latrine .... .3<br>Flush to somewhere else . . .. .4<br>Flush, don't know where . .. . .5<br>Ventilated improved pit latrine (VIP). . .6<br>Pit latrine with slab . . .. .7<br>Pit latrine without slab/open pit . . . 8<br>No facility/bush/field . . . . .9<br>Other (specify)............................96	If 9 or 96, skip to Q418
416	Do you share this toilet facility with other households?	Yes 1	If 0, skip

		No 0	to Q418
417	How many households use this toilet facility? (Write 98 if he/she doesn't know the number of households)		
418	Does your household have electricity?	Yes...................................1<br><br>No....................................0	
419	Does your household have a wall clock?	Yes...................................1<br><br>No....................................0	
420	Does your household have a radio?	Yes...................................1<br><br>No....................................0	
421	Does your household have a television?	Yes...................................1<br><br>No....................................0	
422	Does any member of your household have a mobile telephone?	Yes...................................1<br><br>No....................................0	
423	Does your household have a non-mobile telephone?	Yes...................................1<br><br>No....................................0	
424	Does your household have a refrigerator?	Yes...................................1<br><br>No....................................0	
425	Does your household have a table?	Yes...................................1<br><br>No....................................0	
426	Does your household have chairs?	Yes...................................1<br><br>No....................................0	
427	Does your household have a bed with cotton/sponge/spring mattress?	Yes...................................1<br><br>No....................................0	
428	Does your household have an electric stove for baking enjera or bread (mitad)?	Yes...................................1<br><br>No....................................0	
429	Does your household have a kerosene lamp/pressure lamp?	Yes...................................1<br><br>No....................................0	
430	What type of fuel does your household mainly use for cooking?	Electricity . . . .1<br><br>Liquefied Petroleum Gas (LPG) . . . . 2<br><br>Natural gas/biogas/kerosine.......... . . . . .3<br><br>Charcoal 4	

		Wood . . . . .5<br>Straw/shrubs/grass . . . .6<br>Animal dung . . . . 7<br>No food cooked in household . . .. 95<br>Other (specify)..96	
431	Is the cooking usually done in the house, in a separate building, or outdoors?	In the house . . . . 1<br>In a separate building/room..... . . . . 2<br>Outdoors . . . . 3<br>Other (specify)..96	
432	What is the main material of the floor?	Earth/sand . . . . 1<br>Dung . . . . 2<br>Unpolished wood...................... . . . . 3<br>Polished wood . . ..4<br>Cement . . . . 5<br>Ceramic tiles . . . . 6<br>Carpet . . .. 7<br>Other (specify)......................................96	
433	What is the main material of the roof?	No roof . . . . 1<br>Thatch/leaf/mud . . . . 2<br>Plastic sheets . . .. .3<br>Wood . . . .4<br>Corrugated iron /metal .... 5<br>Cement/concrete . . . . 6<br>Other (specify)...............................96	
434	What is the main material of the exterior walls?	No walls . . . . 1<br>Cane/bamboo ....2<br>Bamboo/wood with mud....... . 3<br>Stone with mud . . . . 4<br>Uncovered adobe (earthen brick)..... 5<br>Cardboard . . . . 6<br>Cement 7	

		Stone with lime/cement . . . . . .8<br>Bricks . . . . .9<br>Cement blocks . . . . .10<br>Covered adobe . . . . 11<br>Other (specify)................96	
435	How many rooms in your household are used for sleeping?		
436	Does any member of your household own a bicycle?	Yes..................................1<br>No...................................0	
437	Does any member of your household own a motorcycle?	Yes..................................1<br>No...................................0	
438	Does any member of your household own an animal-drawn cart?	Yes..................................1<br>No...................................0	
439	Does any member of your household own a car or truck?	Yes..................................1<br>No...................................0	
440	Does any member of your household own any agricultural land?	Yes..................................1<br>No...................................0	If 0, skip to Q445
441	How many hectares of agricultural land do members of your household own?<br><br>1 hectare=10,000 m^2 e.g 100 meter x 100 meter	Yes..................................1<br>No...................................0	
442	What is the annual production of crops/ grains in quintal		
443	Does any member of your household own coffee plants?	Yes..........................1<br>No..........................0	If 0, skip to Q445
444	How many kilograms of coffee do you get on average each year?		
445	Does your household own any livestock, herds, other farm animals, or poultry?	Yes........................1<br>No.........................0	If 0, skip to Q453
446	How many milk cows, oxen or bulls do your household own?		

	If none, enter '0'; If between 1-94, write the exact number; If more than 95, enter '95'; If unknown, enter '98'.		
447	How many horses, donkeys, or mules do your household own?<br><br>If none, enter '0'; If between 1-94, write the exact number; If more than 95, enter '95'; If unknown, enter '98'.		
448	How many camels do your household own?<br><br>If none, enter '0'; If between 1-94, write the exact number; If more than 95, enter '95'; If unknown, enter '98'.		
449	How many goats do your household own?<br><br>If none, enter '0'; If between 1-94, write the exact number; If more than 95, enter '95'; If unknown, enter '98'.		
450	How many sheep do your household own?<br><br>If none, enter '0'; If between 1-94, write the exact number; If more than 95, enter '95'; If unknown, enter '98'.		
451	How many chickens do your household own?<br><br>If none, enter '0'; If between 1-94, write the exact number; If more than 95, enter '95'; If unknown, enter '98'.		
452	How many beehives do your household own?<br><br>If none, enter '0'; If between 1-94, write the exact number; If more than 95, enter '95'; If unknown, enter '98'.		
453	Does any member of your household have a bank or microfinance saving account?	Yes.........................1<br><br>No......... 0	

SECTION 4: CLINICAL BACKGROUND

(Get the information from the TB register)

S.N	Questions and filters	Coding category (Fill)	Skip
501	Type of TB	Bacteriologically confirmed PTB…1<br><br>Clinically confirmed PTB…2<br><br>Clinically confirmed EPTB…3	
502	Type of treatment	New…1<br><br>Retreatment…2<br><br>MDR TB treatment...3	
503	Date of treatment initiation (dd/mm/yyyy) (Ethiopian Calendar)	[_____|_____| ______| <br>dd | mm| yyyy	
504	Duration of TB treatment for the patient (In weeks)		
505	Co-morbid conditions	HIV…1<br><br>DIABETES MELITUS…2<br><br>Asthma…3<br><br>Other (Specify)............96<br><br>No information....98	

SECTION 5: KNOWLEDGE ON TB

S.N	Questions and filters	Coding category (Fill)	Skip
601	Have you ever heard of an illness called TB?	Yes……...……………………1<br><br>No…………………………….0	If no, End here
602	Where did you first learn/hear about TB?<br><br>Record all mentioned.	Newspapers and magazines….1<br><br>Radio….2<br><br>TV….3<br><br>Billboards…4<br><br>Brochures, posters and other printed materials…5<br><br>Health extension workers…6<br><br>Other health workers…7<br><br>Health development armies…8<br><br>Family, friends, neighbors and colleagues…9<br><br>Religious leaders…10<br><br>Teachers…11<br><br>Other (Specify)........96	
603	In your opinion, how serious a disease is TB?	Very serious…1<br><br>Somewhat serious…2<br><br>Not very serious…3	
604	How serious a problem do you think TB is in your community?	Very serious…1<br><br>Somewhat serious…2<br><br>Not very serious…3	
605	What is the cause of TB? (Don't read the options to him/her. Check all the options mention by him/her)<br><br>Probe: Anything else?	Germs….1<br><br>Evil eye….2<br><br>Satan/witchcraft….3<br><br>Other (Specify)...........96<br><br>Don't know…..99	
606	How can a person get TB? (Don't read the options to him/her. Check all the options mention by him/her)	Through the air when coughing or sneezing ….1<br><br>Through sharing utensils….2<br><br>Through touching a person with TB…...3	

	Probe: Any other?	Through food or water…..4<br><br>Through sexual contact with a person with TB….5<br><br>Through mosquito bites….6<br><br>Through drinking un-boiled milk….7<br><br>Exposure to cold….8<br><br>Other (Specify)............96<br><br>Don't know…..99	
607	In your opinion, who can be infected with TB? (Don't read the options to him/her . Check all the options mention by him/her)<br><br>Probe: Any other person?	Anybody…1<br><br>Only poor people…2<br><br>Only homeless people….3<br><br>Only alcoholics…4<br><br>Only drug users…5<br><br>Only people living with HIV/AIDS…6<br><br>Only people who have been in prison…7<br><br>Other (Specify)..............96	
608	What are the body parts affected with TB? (Don't read the options to him/her . Check all the options mention by him/her)<br><br>Probe: Any other part of the body?	Lung….1<br><br>Intestine….2<br><br>Bone….3<br><br>Lymph nodes....4<br><br>Other (Specify).......96<br><br>Don't know…..99	
609	What symptoms will a person with tuberculosis or TB have? (Don't read the options to him/her . Check all the options mention by him/her)<br><br>Probe: Anything else?	Cough........1<br><br>Persistent cough (greater that two weeks)……..…2<br><br>Weight loss……...3<br><br>Poor appetite….4<br><br>Night sweating……...…5<br><br>Chest pain………..…..6<br><br>Fever…………………7<br><br>Blood in the sputum….8<br><br>Shortness of breath….9<br><br>Fatigue….10	

		Swelling.....11<br><br>Other (Specify)..........96<br><br>Don't know…..99	
610	Is TB preventable disease?	Yes…………………………………1<br>No…………...…………………….0<br><br>Don't know…..99	If 0 or 99, skip to Q612
611	What possible TB transmission prevention methods are there?<br><br>(Don't read the options to him/her . Check all the options mention by him/her)<br><br>Probe: Anything else?	Avoiding cough in front of people….1<br><br>Safe disposal of sputum….2<br><br>Ventilation of living room….3<br><br>Avoiding close contact with TB patients….4<br><br>Vaccination for children……5<br><br>Other (Specify)...........96<br><br>Don't know…..99	
612	Can TB be cured?	Yes…………………………1<br>No………………………....0<br><br>Don't know…..99	If 0 or 99, skip to Q614
613	How can someone with TB be cured?	Herbal remedies…1<br><br>Home rest without medicine…2<br><br>Praying…3<br><br>Specific drugs given by health professionals…4<br><br>Other (Specify)........96<br><br>Don't know…..99	
614	What is the duration of your TB treatment? (in months)<br><br>Write '99' if the response is 'I don't know'		
615	Do you think that you are well informed about TB (on its prevention and control)?	Yes……………………………1<br>No………………...……….0	
616	Do you wish to get more information about TB?	Yes…………………………………………1<br>No……………………………………….0	If 0, Skip to Q701
617	What are the sources of information that you think can most effectively reach people like you with information on TB? (Please choose the three most effective sources.)	Newspapers and magazines…1<br><br>Radio…2<br><br>TV…3<br><br>Billboards…4	

		Brochures, posters and other printed materials…5<br><br>Health extension workers…6<br><br>Other health workers…7<br><br>Health development armies…8<br><br>Family, friends, neighbours and colleagues…9<br><br>Religious leaders…10<br><br>Teachers…11<br><br>Other (Specify)...........96	
618	Have you ever heard about multidrug resistant TB?	Yes……………………………1<br>No…………………....……….0	If 0, Skip to Q701
619	What do you know about multidrug resistant TB?<br><br><br>Probe: Anything else?	It is created when TB patients do not take anti-TB drugs regularly………1<br><br>It is a dangerous form of TB…2<br><br>Can be transmitted to family and friends….3<br><br>Its treatment lasts for at least 2 years….4<br><br>Other (specify)__________________5<br><br>Can not explain multidrug resistant TB…6	

SECTION 6: ATTITUDE AND STIGMA RELATED QUESTIONS

(Read the options to the participant and inform him/her to select one option only)

S.N	Questions and filters	Coding category (Fill)	Skip
701	Being a TB patient, others would think less of you.	Strongly disagree...........1<br>Disagree.........................2<br>Indifferent......................3<br>Agree.............................4<br>Strongly agree.................5	
702	Being a TB patient, you would be ashamed/embarrassed.	Strongly disagree...........1<br>Disagree.........................2<br>Indifferent......................3<br>Agree.............................4<br>Strongly agree.................5	
703	Being a TB patient, others would avoid you.	Strongly disagree...........1<br>Disagree.........................2<br>Indifferent......................3<br>Agree.............................4<br>Strongly agree.................5	
704	*Do you have partner?*	*Yes………………………………………1*<br>*No……………………………………….0*	*If 1, skip to Q706*
705	*Only for the respondent who has no partner - Being a TB patient, you would have a problem of finding a partner for marriage even after cure.*	*Strongly disagree...........1*<br>*Disagree.........................2*<br>*Indifferent......................3*<br>*Agree.............................4*<br>*Strongly agree.................5*	
706	*Only for the respondent who has partner- Being a TB patient, your partner would refuse to have sex with you.*	*Strongly disagree...........1*<br>*Disagree.........................2*<br>*Indifferent......................3*<br>*Agree.............................4*<br>*Strongly agree.................5*	*Those who responded 0 to Q704 shall not respond to this question*
707	Being a TB patient, you would be asked to stay away from a social	Strongly disagree...........1	

	group.	Disagree.........................2<br>Indifferent......................3<br>Agree.............................4<br>Strongly agree.................5	
708	Being a TB patient, you would not disclose even to a confidant (Somebody to whom secrets are usually told).	Strongly disagree...........1<br>Disagree.........................2<br>Indifferent......................3<br>Agree.............................4<br>Strongly agree.................5	
709	Being a TB patient, you would think less of yourself	Strongly disagree...........1<br>Disagree.........................2<br>Indifferent......................3<br>Agree.............................4<br>Strongly agree.................5	
710	Being a TB patient, others would think less of your family	Strongly disagree...........1<br>Disagree.........................2<br>Indifferent......................3<br>Agree.............................4<br>Strongly agree.................5	
711	Do you have children?	Yes...1<br>No..0	If '0' skip to 713
712	Being a TB patient, it would be a problem for your children	Strongly disagree...........1<br>Disagree.........................2<br>Indifferent......................3<br>Agree.............................4<br>Strongly agree.................5	
713	Being a TB patient, you would be less likely to find a job.	Strongly disagree...........1<br>Disagree.........................2<br>Indifferent......................3<br>Agree.............................4<br>Strongly agree.................5	

714	Being a TB patient, you would lose your job.	Strongly disagree...........1<br>Disagree.........................2<br>Indifferent......................3<br>Agree.............................4<br>Strongly agree.................5	

SECTION 7: PERCEPTION AS TB PATIENT ON OTHER TB PATIENTS, ON TB IN GENERAL, AND SATISFACTION ON HEALTH SERVICES

S.N	Questions and filters	Coding category (Fill)	Skip
801	Family members are cooperative towards me. (Read the options to the participant and inform him/her to select one option only)	Strongly disagree...........1<br>Disagree.........................2<br>Indifferent......................3<br>Agree.............................4<br>Strongly agree................5	
802	I have increased sadness. (Read the options to the participant and inform him/her to select one option only)	Strongly disagree...........1<br>Disagree.........................2<br>Indifferent......................3<br>Agree.............................4<br>Strongly agree.................5	
803	Utensils are separated for me. (Read the options to the participant and inform him/her to select one option only)	Strongly disagree...........1<br>Disagree.........................2<br>Indifferent......................3<br>Agree.............................4<br>Strongly agree.................5	
804	I have fear of reduction of family income. (Read the options to the participant and inform him/her to select one option only)	Strongly disagree...........1<br>Disagree.........................2<br>Indifferent......................3<br>Agree.............................4<br>Strongly agree.................5	
805	Threat of loss of job/wages. (Read the options to the participant and inform him/her to select one option only)	Strongly disagree...........1<br>Disagree.........................2<br>Indifferent......................3<br>Agree.............................4<br>Strongly agree.................5	
806	Most people behave differently. (Read the options to the participant and inform him/her to select one option only)	Strongly disagree...........1<br>Disagree.........................2<br>Indifferent......................3	

		Agree.............................4<br><br>Strongly agree................5	
807	Feel isolated within the family. (Read the options to the participant and inform him/her to select one option only)	Strongly disagree...........1<br><br>Disagree.........................2<br><br>Indifferent......................3<br><br>Agree.............................4<br><br>Strongly agree................5	
808	Family members avoid me. (Read the options to the participant and inform him/her to select one option only)	Strongly disagree...........1<br><br>Disagree.........................2<br><br>Indifferent......................3<br><br>Agree.............................4<br><br>Strongly agree................5	
809	Which statement is closest to your feeling about people with TB disease?<br><br>(Read the options to the participant and inform him/her to select one option only)	I feel compassion and desire to help….1<br><br>I feel compassion but I tend to stay away from these people.…2<br><br>It is their problem and I do not want to get TB by trying to help them…3<br><br>I fear them because they may re-infect me.…4<br><br>I have no particular feeling.…5<br><br>Other (Specify)........96	
810	The health care providers are supportive and respectful of people who have TB. (Read the options to the participant and inform him/her to select one option only)	Strongly disagree...........1<br><br>Disagree.........................2<br><br>Indifferent......................3<br><br>Agree.............................4<br><br>Strongly agree................5	
811	Before coming for diagnosis/ treatment, you expected that the providers would be supportive and respectful of people who have TB. (Read the options to the participant and inform him/her to select one option only)	Strongly disagree...........1<br><br>Disagree.........................2<br><br>Indifferent......................3<br><br>Agree.............................4<br><br>Strongly agree................5	
812	Most people in your community believe that they will be treated supportively and respectfully by health care providers if they have TB. (Read the options to the participant and inform him/her to select one	Strongly disagree...........1<br><br>Disagree.........................2	

	option only)	Indifferent......................3<br>Agree..............................4<br>Strongly agree.................5	
813	You are happy with the service you are getting from this facility. (Read the options to the participant and inform him/her to select one option only)	Strongly disagree...........1<br>Disagree.........................2<br>Indifferent......................3<br>Agree..............................4<br>Strongly agree.................5	
814	How did you find the level of service you are getting from this facility comparing to your expectations before getting the health service in this health facility? (Read the options to the participant and inform him/her to select one option only)	Better than you expected…1<br>About what you expected…2<br>Not as good as you expected…3	
815	Health care workers answered all of your questions concerning TB. (Read the options to the participant and inform him/her to select one option only)	Strongly disagree...........1<br>Disagree.........................2<br>Indifferent......................3<br>Agree..............................4<br>Strongly agree.................5	
816	You are satisfied with health post/health center schedule. (Read the options to the participant and inform him/her to select one option only)	Strongly disagree...........1<br>Disagree.........................2<br>Indifferent......................3<br>Agree..............................4<br>Strongly agree.................5	
817	You are satisfied with waiting time in the health post/health center. (Read the options to the participant and inform him/her to select one option only)	Strongly disagree...........1<br>Disagree.........................2<br>Indifferent......................3<br>Agree..............................4<br>Strongly agree.................5	
818	You are satisfied with the availability of drugs at the health center. (Read the options to the participant and inform him/her to select one option only)	Strongly disagree...........1<br>Disagree.........................2<br>Indifferent......................3<br>Agree..............................4	

		Strongly agree.................5	
819	Do you have TB treatment supporter?	Yes...1<br><br>No...0	If ‘0’ skip to 901
820	You are satisfied with your TB treatment supporter. (Read the options to the participant and inform him/her to select one option only)	Strongly disagree...........1<br><br>Disagree.........................2<br><br>Indifferent......................3<br><br>Agree..............................4<br><br>Strongly agree.................5	

SECTION 8: PRACTICE RELATED QUESTIONS

S.N	Questions and filters	Coding category (Fill)	Skip
901	How long have you stayed with the signs and symptoms of TB (Cough, fever, night sweats, chest pain, hemoptysis, lump, etc) until you first sought care in health institution? (in days starting from onset of symptoms)		Skip to '903' if it is less than 30 days
902	What factors made you delay seeking treatment for symptoms that led to the diagnosis of TB? (More than one answer possible)	Not aware of the severity of my symptoms…1<br><br>Fear of rejection/ losing my job…2<br><br>Fear that treatment is expensive…3<br><br>Lack of time…4<br><br>Difficult access to health center/transportation issues…5<br><br>Not having a previous satisfactory experience with the health system…6<br><br>I feel that there was no delay in seeking treatment for symptoms that led to the diagnosis of TB….7<br><br>Other (specify)……………..…96	
903	How long did it take since you first sought health attention until diagnosis of TB? (in days)		
904	How long did it take from diagnosis of TB to beginning treatment? (in days)		
905	Did you seek any advice from drug vendors, traditional or spiritual healers before visiting health institution?	Yes……..1<br><br>No………0	If 0, skip to Q907
906	Which healers did you visit before visiting health institution? (More than one answer possible)	Private drug vendors…………1<br><br>Religious leaders………..2<br><br>Traditional healers…………3<br><br>Other (specify)…………..…96	
907	Were you first seen by Health Extension Worker for the current complaint and referred by HEW to health facility?	Yes……………………………1<br>No…………………………….0	
908	Health facility you first diagnosed with TB.	In the same health facility…………1<br><br>Referred from government hospital…………2<br><br>Referred from government Health Center……..3<br><br>Referred from private health facility …………4<br><br>Referred from NGO health facility…….5<br><br>Other (specify)…………..…96	
909	Who do you think can better reduce the delay in the diagnosis and treatment for	The patient…1	

	TB? (More than one option possible)	The family…2<br>The health system…3<br>The government…4<br>Other (specify)……………..…96	
910	How long have you taken the TB medicine? (in weeks)		
911	Where do you currently take the TB drugs on daily basis	In the health center…..1<br>In the health post…..2<br>At home….3<br>Other (specify)……..…96	
912	How far is the HC or HP from your house? (In minutes)		
913	How frequently do you visit the health facility for your current treatment?	Every day..................1<br>Every week............2<br>Every 2 weeks.......3<br>Every month.......4<br>Every 2 months......5<br>Every 3 months....6<br>Every more than 3 months.....7	
914	Are you taking the drugs without interruption?	Yes……………………1<br>No……...…………….0	If 1, Skip to Q917
915	If you have interrupted the drugs, how long have you interrupted? (in week)		
916	What were the reasons for interrupting to take the TB drugs? (More than one option possible)	There was no drug at the health facility….1<br>I developed side effects of the drugs…2<br>I went to traditional healers/holly water…3<br>Health facility is too far……4<br>*I have been outside my residence area for social reasons....5*<br>*I was too weak to come to the health facility/there was no one who can collect the drugs for me....6*<br>Other (specify)……..…96	
917	Is there anyone who observes you when you take the drugs daily?	Yes……………………1<br>No…………………….0	If 0, Skip to Q919
918	Who observe you when you take the drugs daily?	Health professionals at health center…………………1<br>Health extension workers……2<br>TB treatment supporter who is family member…….3	

		TB treatment supporter who is neighbour….4<br><br>TB treatment supporter who is community figure…5<br><br>Other (specify)………………...96	
919	Have you informed any family member that you have TB?	Yes………………………1<br>No……………………….0	If 0, Skip to Q921
920	Among the family members, who did you inform that you have TB? (More than one option possible)	Husband…..…………………1<br><br>Wife………………………..2<br><br>Sister/ Brother…………….3<br><br>Son/ Daughter……………4<br><br>Other (specify)…..……..96	
921	Have you informed anyone else who is not family member that you have TB?	Yes……………………1<br>No…………………….0	If 0, Skip to Q923
922	Among people who are not family members, who did you inform that you have TB?	Religious leaders…………..1<br><br>Neighbours…………..2<br><br>Colleagues/friends at work place……..3<br><br>Friends who are not working with me……..4<br><br>Other (specify)…………..…..96	
923	Is the health extension worker in your kebele communicated that you have TB?	Yes…………………1<br>No………………….0	If 0, skip to Q925
924	Who communicated the HEW about your TB?	Professionals at the health center…..1<br><br>Myself by taking a note written by the professionals at the health center….2<br><br>Family member……3<br><br>TB treatment supporter who is not family member…4<br><br>Other (Specify)…………………..96	
925	Are you eating more and a variety of food after you knew that you have TB?	Yes……………………….1<br><br>No…………………………0	
926	Do you open windows/doors in your house after you knew that you have TB?	Yes……………………….1<br><br>No…………………………0	
927	Do you open windows/doors in your work place after you knew that you have TB?	Yes……………………….2<br><br>No…………………………1<br><br>I have no work outside home…..0	
928	Is your weight checked regularly?	Yes……………………….1	

		No……………………0	
929	Were there times that the health professionals informed you that your weight is lower than expected?	Yes……………………1<br>No……………………0	If 0, Skip to Q931
930	If your weight was lower than expected, did you receive any nutritional support from the health facilities?	Yes……………………1<br>No……………………0	
931	Do you cover your mouth when coughing?	Yes……………………1<br>No……………………0	
932	Have you informed family members to go to the health institutions for investigation?	Yes……………………1<br>No………………………0	If 0, Skip to Q942
933	Did they go for investigation?	Yes……………………1<br>No………………………0	If 0, Skip to Q942
934	How many family members were investigated? *Write 98 if he/she has no family*		
935	How many children under-five years were investigated? *Write '0' if there was no under-five years old child who is investigated Write 98 if he/she has no children*		
936	How many people age 5 years to less than 18 years were investigated?<br>*Write '0' if there was no person age 5 years to less than 18 years old who is investigated*<br>*Write 98 if he/she has no person age 5-18 years*		
937	How many adults (18 and more years old) were investigated? *Write '0' if there was no adult who is investigated*<br>*Write 98 if he/she has no adult*		
938	Were there any family member diagnosed to have TB after the investigation?	Yes……………………1<br>No……………………0	If 0, Skip to Q942
939	How many children under-five years of age were diagnosed to have TB after the investigation?<br>*Write '0' if there was no under-five year old child who is diagnosed to have TB*		
940	How many people age 5 years to less than 18 years were diagnosed to have TB after the investigation?<br>*Write '0' if there was no person age 5 years to less than 18 years who is diagnosed to have TB*		
941	How many adults (18 and more years) were diagnosed to have TB after the investigation? *Write '0' if there was no adult who is diagnosed to have TB*		
942	Are there children whose age is under-five years in your family?	Yes……………………1<br>No………………………0	If 0, Skip to Q948
943	Number of children under-five years of age in the family		
944	Did the children under-five years old get preventive therapy for TB?	Yes……………………1<br>No………………………0	If 0, Skip to Q946

945	How many children under-five years old get preventive therapy for TB?		
946	Is there a child whose age is under one year in the family?	Yes………………………1<br><br>No…………………………0	If 0, Skip to Q948
947	Is he/she vaccinated for TB	Yes………………………1<br><br>No…………………………0	
948	Are there family members in the house who have HIV?	*Yes………..........……………..2*<br><br>*No………….......……………1*<br><br>*I don't know......................0.*	*If 1 or 0, Skip to Q950*
949	Did these family members who have *HIV* get preventive therapy for TB?	Yes………………………1<br><br>No…………………………0	
950	Did any of the following happen on you after developing TB disease?<br><br>(Check all that apply.)	Lost job…1<br><br>Reduction in income…2<br><br>Divorce…3<br><br>Interrupted school…4<br><br>Nothing…5<br><br>Other (Specify).....96	

End of questions

Thank you very much for your cooperation
