# Supplementary material for: Knowledge, attitudes, and practices related to TB among the general population of Ethiopia: Findings from a national cross-sectional survey
Source: PLoS One. 2019 Oct 28;14(10):e0224196. doi: 10.1371/journal.pone.0224196 (PMC6816561; doi:10.1371/journal.pone.0224196)
Supplement: S3 Table — (PDF) [file pone.0224196.s003.pdf]

Supporting table 3 Knowledge about cause and risk of TB in Ethiopia, 2017

**Variables**		General population N=1,783		TB patients N=844		Families of TB patients N=836		Total N= 3,463	
		N	%	N	%	N	%	N	%
Have you ever heard of TB	Yes	1,668	93.6	823	97.5	815	97.5	3,306	95.5
	No	115	6.5	21	2.5	21	2.5	157	4.5
Cause of TB	Germs	502	28.2	224	26.6	260	31.1	986	28.5
	Evil eye	6	0.3	4	0.5	4	0.5	14	0.4
	Satan/witchcraft	22	1.2	7	0.8	8	1.0	37	1.1
	Other causes	347	19.5	164	19.4	179	21.4	690	19.9
	Don't know	810	45.4	436	51.7	380	45.5	1,626	47.0
How can a person get TB?	Through air (coughing and sneezing)	1,238	69.4	586	69.4	600	71.8	2,424	70.0
	Sharing utensils	655	36.7	332	39.3	329	39.4	1,316	38.0
	Touching a person with TB	215	12.1	96	11.4	115	13.8	426	12.3
	Through food or water	245	13.7	115	13.3	120	14.4	480	13.9
	Sexual contact TB case	141	7.9	65	7.7	69	8.3	275	7.9
	Mosquito bites	29	1.6	11	1.3	10	1.2	50	1.4
	Drinking raw milk	83	4.7	30	3.6	48	5.7	161	4.7
	Exposure to cold	165	9.3	110	13.0	99	11.8	374	10.8
	Others	48	2.7	7	0.8	15	1.8	70	2.0
	Don't know	226	12.7	131	15.5	115	13.8	472	13.6
Who can be infected with TB?	Anybody	1,291	72.4	669	79.3	667	79.8	2,627	75.9
	Only poor people	249	14.0	148	17.5	134	16.0	531	15.3
	Only homeless people	148	8.3	73	8.7	71	8.5	292	8.4
	Only alcoholics	179	10.0	75	8.9	72	8.6	326	9.4
	Only drug users	115	6.5	59	7.0	43	5.1	217	6.3
	Only those living with HIV	115	6.5	47	5.6	48	5.7	210	6.1
	Only those who have been in prison	39	2.2	11	1.0	4	0.5	54	1.6
	Others	137	7.7	44	5.2	35	4.2	216	6.2
Body parts affected with TB	Lung	1,344	75.4	728	86.3	699	83.6	2,771	80.0
	Intestine	118	6.6	86	10.2	86	10.3	290	8.4
	Bone	342	19.2	236	28.0	229	27.4	807	23.3
	Lymph nodes	101	5.7	90	10.7	81	9.7	272	7.9
	Others	81	4.5	24	2.8	17	2.0	122	3.5
	Don't know	237	13.3	57	6.8	88	10.5	382	11.0
Symptoms of TB	Cough	1,333	74.8	690	81.8	690	82.5	2,713	78.3
	Persistent cough	396	22.2	202	23.9	190	22.7	788	22.8
	Weight loss	677	38.0	361	42.8	373	44.6	1,411	40.8
	Poor appetite	336	18.8	249	29.5	238	28.5	823	23.8
	Night sweating	263	14.8	193	22.9	192	23.0	648	18.7
	Chest pain	250	14.0	187	22.2	159	19.0	596	17.2
	Fever	250	14.0	178	21.1	165	19.7	593	17.1
	Blood in the sputum	255	14.3	114	13.5	96	11.5	465	13.4
	Shortness of breath	110	6.2	62	7.4	45	5.4	217	6.3
	Fatigue	187	10.5	152	18.0	98	11.7	437	12.6
	Swelling	96	5.4	87	10.3	68	8.1	251	7.3
	Others	28	1.6	21	2.5	8	1.0	57	1.7
	Don't know	123	6.9	13	1.5	27	3.2	163	4.7
Is TB preventable	Yes	1,347	75.6	627	74.3	664	79.4	2,638	76.2
	No	114	6.4	65	7.7	64	7.7	243	7
	Do not know	207	11.6	131	15.5	87	10.4	425	12.3
Prevention methods	Avoiding cough in front of people	923	51.8	455	53.9	495	59.2	1,873	54.1
	Safe disposal of sputum	425	23.8	217	25.7	210	25.12	852	24.60
	Ventilation of living room	300	16.8	137	16.2	171	20.45	608	17.56
	Avoiding close contact with TB patients	524	29.4	268	31.8	273	32.67	1,065	30.8
	Vaccination of children	97	5.4	33	3.9	40	4.8	170	4.9
	Others	179	10.0	55	6.5	51	6.1	285	8.2
	Don't know	112	6.3	50	5.9	44	5.3	206	6.0
TB can be cured	Yes	1,506	84.5	788	93.4	784	93.8	3,078	88.9
	No	54	3.0	3	0.4	6	0.7	63	1.8
	I don't know	108	6.1	32	3.8	25	3.0	165	4.8
How can someone with TB be cured?	Specific drugs given by health workers	1,439	80.7	761	90.2	753	90.1	2,953	85.3
	Home rest without medicine	45	2.5	18	2.1	21	2.5	84	2.4
	Praying	4	0.2	2	0.2	1	0.1	7	0.2
	Others*	3	0.2	2	0.2	0	0.0	5	0.2
	Don't know	15	0.8	5	0.6	9	1.1	29	0.8
