# Supplementary material for: Knowledge, attitudes, and practices related to TB among the general population of Ethiopia: Findings from a national cross-sectional survey
Source: PLoS One. 2019 Oct 28;14(10):e0224196. doi: 10.1371/journal.pone.0224196 (PMC6816561; doi:10.1371/journal.pone.0224196)
Supplement: S4 Table — (PDF) [file pone.0224196.s004.pdf]

Supporting table 4 Knowledge and source of information about TB in Ethiopia, 2017

Variables		General population		Families of TB patients		TB patients	
		N	%	N	%	N	%
Ever heard of TB		1668	93.6	815	97.5	823	97.5
First source of Information about TB	Newspapers and magazines	26	1.6	11	1.3	8	1.0
	Radio	605	36.3	274	33.6	235	28.6
	TV	586	35.1	273	33.5	227	27.6
	Billboards	24	1.4	13	1.6	10	1.2
	Brochures, posters and other print medias	65	3.9	49	6.0	42	5.1
	HEWs	507	30.4	223	27.4	227	27.6
	Other Health Workers	367	22	261	32.0	272	33.0
	HDAs	128	7.7	79	9.7	79	9.6
	Family, friends, neighbors and colleagues	891	53.4	442	54.2	450	54.7
	Religious leaders	62	3.7	29	3.2	24	2.9
	Teachers	175	10.5	86	10.6	70	8.5
	Others	11	0.6	11	1.3	5	0.6
Well informed on TB		460	27.6	268	32.9	240	29.2
Wished to get more information on TB		1547	92.7	744	91.3	746	90.6
Perceived to be effective information source	Newspapers and magazines	89	5.8	33	4.4	34	4.6
	Radio	862	55.7	414	55.6	399	53.5
	TV	809	52.3	348	46.8	331	44.4
	Billboards	31	2.0	16	2.2	17	2.3
	Brochures, posters and other print medias	135	8.7	71	9.5	56	7.5
	HEWs	986	63.7	450	60.5	455	61.0
	Other Health Workers	676	43.7	405	54.4	413	55.4
	HDAs	260	16.8	125	16.8	126	16.9
	Family, friends, neighbors and colleagues	382	24.7	206	27.7	220	29.5
	Religious leaders	198	12.8	80	10.8	98	13.1
	Teachers	184	11.9	79	10.6	79	10.6
	Others	29	1.9	5	0.7	10	1.3
Heard of multidrug resistance TB		349	20.9	206	25.3	238	28.9
What do you know about multidrug resistant TB?	Created when TB patients don't take anti-TB drugs regularly	266	76.2	157	76.2	180	75.6
	It is dangerous form of TB	138	39.5	95	46.1	79	33.2
	Can be transmitted to family and friends	50	14.3	22	10.7	17	7.1
	Its treatment lasts for at least 2 years	33	9.5	19	9.2	22	9.2
	Not curable/Getting TB disease again	4	1.1	21	10.2	3	1.3
	Cannot explain	34	9.7	157	76.2	34	14.3
