# Supplementary material for: Knowledge, attitudes, and practices related to TB among the general population of Ethiopia: Findings from a national cross-sectional survey
Source: PLoS One. 2019 Oct 28;14(10):e0224196. doi: 10.1371/journal.pone.0224196 (PMC6816561; doi:10.1371/journal.pone.0224196)
Supplement: S5 Table — (PDF) [file pone.0224196.s005.pdf]

**Supporting table 5 Factors associated with knowledge about TB in the general population in Ethiopia, 2017**

| Variables | | Knowledge High # (%) | Knowledge Low # (%) | COR (95% CI) | AOR (95%CI) |
|---|---|---|---|---|---|
| Gender | Male | 424(54.2) | 359(45.8) | 1.36(1.12-1.65) | 1.38(1.12-1.71)* |
| | Female | 411(46.4) | 474(53.6) | 1 | 1 |
| Education | Not able to read and write | 181(38.3) | 292 (61.7) | 1 | 1 |
| | Read and write only | 49(41.5) | 69(58.5) | 1.15(0.76-1.73) | 1.02(0.65-1.59) |
| | Primary | 236(48.6) | 250(51.4) | 1.52(1.18-1.97) | 1.33(1.0-1.77)* |
| | Secondary | 226(58.1) | 163(41.9) | 2.24(1.7-2.94) | 1.85(1.36-2.52)* |
| | Above secondary | 143(70.8) | 59(29.2) | 3.91(2.74-5.58) | 3.17(2.11-4.75)* |
| Wealth | Lowest | 100(32.7) | 206(67.3) | 0.25(0.18-0.35) | 0.33(0.22-0.49)* |
| | Second | 148(46.0) | 174(54.0) | 0.44(0.32-0.6) | 0.48(0.33-0.71)* |
| | Third | 154(45.7) | 183(54.3) | 0.44(0.32-0.59) | 0.49(0.35-0.71)* |
| | Fourth | 198(57.2) | 148(42.8) | 0.7(0.51-0.94) | 0.76(0.54-1.07) |
| | Highest | 235(65.8) | 122(34.2) | 1 | 1 |
| Setting | Rural | 308(41.7) | 431(58.3) | 0.55(0.45-0.66) | 0.82(0.64-1.04) |
| | Urban | 527(56.7) | 402(43.3) | 1 | 1 |
| Region | Oromia | 205(62.3) | 124(37.7) | 1 | 1 |
| | Amhara | 96(28.7) | 239(71.3) | 0.24(0.18-0.34) | 0.16(0.11-0.23)* |
| | SNNP | 161(50.6) | 157(49.4) | 0.62(0.45-0.85) | 0.48(0.34-0.67)* |
| | Tigray | 95(53.7) | 82(46.3) | 0.7(0.48-1.02) | 0.55(0.37-0.81)* |
| | Benshangul Gumuz | 31(34.8) | 58(65.2) | 0.32(0.2-0.53) | 0.29(0.18-0.49)* |
| | Gambella | 45(54.9) | 37(45.1) | 0.74(0.45-1.2) | 0.76(0.45-1.27) |
| | Addis Ababa | 119(68.8) | 54(31.2) | 1.33(0.9-1.97) | 0.68(0.44-1.06) |
| | Dire Dawa | 45(55.6) | 36(44.4) | 0.76(0.46-1.24) | 0.47(0.28-0.8)* |
| | Harari | 38(45.2) | 46(54.8) | 0.5(0.31-0.81) | 0.34(0.2-0.57)* |

*P<0.05, The study participants were grouped as having high and low knowledge score using the mean knowledge score as a cut-off point.
