# Supplementary material for: Knowledge, attitudes, and practices related to TB among the general population of Ethiopia: Findings from a national cross-sectional survey
Source: PLoS One. 2019 Oct 28;14(10):e0224196. doi: 10.1371/journal.pone.0224196 (PMC6816561; doi:10.1371/journal.pone.0224196)
Supplement: S6 Table — (PDF) [file pone.0224196.s006.pdf]

Supporting table 6 Factors associated with knowledge about TB among families of TB patients in Ethiopia, 2017

Variables		Knowledge High # (%)	Knowledge Low # (%)	COR (95% CI)	AOR (95%CI)
Gender	Male	176(48.6)	186(51.4)	1.29(0.97-1.72)	1.15(0.83-1.59)
	Female	164(42.3)	224(57.7)	1	1
Education	Not able to read and write	86(37.6)	143(62.4)	1	1
	Read and write only	20(41.7)	28(58.3)	1.19(0.63-2.24)	1.76(0.87-3.55)
	Primary	92(44.4)	115(55.6)	1.33(0.91-1.95)	1.49(0.96-2.31)
	Secondary	84(50.3)	83(49.7)	1.68(1.12-2.52)	2.04(1.27-3.29)*
	Above secondary	58(58.6)	41(41.4)	2.35(1.45-3.81)	2.88(1.63-5.1)*
Wealth	Lowest	53(39.0)	83(61.0)	0.6(0.38-0.95)	0.8(0.43-1.5)
	Second	61(41.5)	86(58.5)	0.67(0.43-1.05)	0.74(0.43-1.29)
	Third	59(40.4)	87(59.6)	0.64(0.41-1.0)	0.71(0.42-1.19)
	Fourth	84(52.5)	76(47.5)	1.04(0.67-1.61)	1.0(0.63-1.63)
	Highest	83(51.6)	78(48.4)	1	1
Setting	Rural	104(39.1)	162(60.9)	0.68(0.5-0.91)	0.71(0.47-1.07)
	Urban	236(48.8)	248(51.2)	1	1
Region	Oromia	82(69.5)	36(30.5)	1	1
	Amhara	39(25.0)	117(75.0)	0.15(0.09-0.25)	0.09(0.05-0.16)*
	SNNP	60(38.0)	98(62.0)	0.27(0.16-0.45)	0.23(0.14-0.39)*
	Tigray	37(44.0)	47(56.0)	0.35(0.19-0.62)	0.22(0.12-0.43)*
	Benshangul Gumuz	15(35.7)	27(64.3)	0.24(0.12-0.51)	0.16(0.07-0.36)*
	Gambella	8(32.0)	17(68.0)	0.21(0.08-0.52)	0.17(0.06-0.46)*
	Addis Ababa	52(61.9)	32(38.1)	0.71(0.4-1.29)	0.41(0.21-0.79)*
	Dire Dawa	25(59.5)	17(40.5)	0.65(0.31-1.34)	0.32(0.14-0.71)*
	Harari	22(53.7)	19(46.3)	0.51(0.25-1.05)	0.35(0.16-0.77)*

*P<0.05, The study participants were grouped as having high and low knowledge score using the mean knowledge score as a cut-off point.
