# Supplementary material for: Knowledge, attitudes, and practices related to TB among the general population of Ethiopia: Findings from a national cross-sectional survey
Source: PLoS One. 2019 Oct 28;14(10):e0224196. doi: 10.1371/journal.pone.0224196 (PMC6816561; doi:10.1371/journal.pone.0224196)
Supplement: S7 Table — (PDF) [file pone.0224196.s007.pdf]

Supporting table 7 Factors associated with knowledge about TB among TB patients in Ethiopia, 2017

Variables		Knowledge High # (%)	Knowledge Low # (%)	COR (95% CI)	AOR (95%CI)
Gender	Male	248(51.7)	232(48.3)	1.43(1.08-1.88)	1.34(0.97-1.84)
	Female	147(42.9)	196(57.1)	1	1
Education	Not able to read and write	116(40.8)	168(59.2)	1	1
	Read and write only	24(63.2)	14(36.8)	2.48(1.23-5.0)	2.14(0.98-4.68)
	Primary	125(49.2)	129(50.8)	1.4(1.0-1.97)	1.3(0.88-1.93)
	Secondary	84(51.5)	79(48.5)	1.54(1.05-2.27)	1.65(1.03-2.63)*
	Above secondary	46(54.8)	38(45.2)	1.75(1.07-2.86)	2.11(1.16-3.85)*
Wealth	Lowest	61(38.6)	97(61.4)	0.54(0.35-0.84)	0.53(0.29-0.98)*
	Second	84(51.2)	80(48.8)	0.9(0.58-1.38)	0.77(0.43-1.36)
	Third	84(48.6)	89(51.4)	0.81(0.53-1.24)	0.7(0.41-1.17)
	Fourth	77(47.2)	86(52.8)	0.77(0.5-1.18)	0.74(0.45-1.2)
	Highest	89(53.9)	76(46.1)	1	1
Setting	Rural	150(44.1)	190(55.9)	0.77(0.58-1.0)	0.6(0.41-0.89)*
	Urban	245(50.7)	238(49.3)	1	1
Region	Oromia	129(76.8)	39(23.2)	1	1
	Amhara	35(20.6)	135(79.4)	0.08(0.05-0.13)	0.05(0.03-0.09)*
	SNNP	77(47.2)	86(52.8)	0.27(0.17-0.43)	0.29(0.18-0.47)*
	Tigray	37(43.0)	49(57.0)	0.23(0.13-0.4)	0.16(0.09-0.29)*
	Benshangul Gumuz	19(45.2)	23(54.8)	0.25(0.12-0.51)	0.2(0.09-0.42)*
	Gambella	12(42.9)	16(57.1)	0.23(0.1-0.52)	0.19(0.08-0.47)*
	Addis Ababa	44(53.7)	38(46.3)	0.35(0.2-0.62)	0.18(0.09-0.34)*
	Dire Dawa	21(50.0)	21(50.0)	0.3(0.15-0.61)	0.17(0.08-0.38)*
	Harari	21(50.0)	21(50.0)	0.3(0.15-0.61)	0.22(0.1-0.48)*

∗P<0.05, the study participants were grouped as having high and low knowledge score using the mean knowledge score as a cut-off point.
