# Supplementary material for: Knowledge, attitudes, and practices related to TB among the general population of Ethiopia: Findings from a national cross-sectional survey
Source: PLoS One. 2019 Oct 28;14(10):e0224196. doi: 10.1371/journal.pone.0224196 (PMC6816561; doi:10.1371/journal.pone.0224196)
Supplement: S8 Table — (PDF) [file pone.0224196.s008.pdf]

Supporting table 8 The distribution of study population by regions in Ethiopia

Region	Zone	General population #	Families of TB patients #	TB patients #
Oromia	West Hararge	90	42	42
	North Shewa	88	45	46
	East Shewa	95	39	39
	West Guji	84	43	42
	Total	357	169	169
Amhara	North Shewa	89	42	42
	West Gojjam	89	43	42
	North Wollo	90	42	47
	Gondar City	89	42	43
	Total	357	169	174
SNNP	Sidama	90	44	44
	Segen area	88	44	44
	Sheka	88	43	43
	Siltie	90	44	43
	Total	356	175	174
Tigray	Western Tigray	89	42	42
	Eastern Tigray	89	42	44
	Total	178	84	86
Benshangul Gumuz	Kamashi	89	43	42
Gambella	Agnuak	90	28	30
Addis Ababa	Bole	44	21	21
	Akaki Kality	44	21	21
	Gullelle	45	21	21
	Addis Ketema	45	21	21
	Total	178	84	84
Dire Dawa	Dire Dawa	89	42	43
Harari	Harari	89	42	42
Setting	Rural	807	313	221
	Urban	976	523	623
Total		1783	836	844
