# Supplementary material for: Knowledge, attitudes, and practices related to TB among the general population of Ethiopia: Findings from a national cross-sectional survey
Source: PLoS One. 2019 Oct 28;14(10):e0224196. doi: 10.1371/journal.pone.0224196 (PMC6816561; doi:10.1371/journal.pone.0224196)
Supplement: S1 File — (PDF) [file pone.0224196.s009.pdf]

INTRODUCTION AND CONSENT

PARTICIPANT INFORMATION SHEET

TITLE OF THE RESEARCH: KAP related to TB among the general population, current TB patients, and families of current TB patients in Ethiopia

Background and purpose

USAID supports TB program in Ethiopia. This is a request for you to participate in a study that intends to assess the KAP of the general population, TB patients, and family members of TB patients regarding TB in nine regions of Ethiopia. The KAP data that will be produced will provide TB program managers the fundamental information needed to make strategic decisions regarding TB control. USAID/CTB Ethiopia contracted ABH Services PLC to conduct the KAP study.

What does the study entail?

We will ask you questions about some background information including socio-economic background, about your KAP related to TB. The questions will be asked by trained data collectors of ABH Services PLC. The interview may last about an hour.

Potential benefits and harm

By participating in the study the data generated will help to inform TB program managers about the fundamental information needed to make strategic decisions regarding the control of TB. There is no harm/disadvantage if you participate in this study except that it takes some of your time. Since the interview takes only about an hour and since there will not be significant transportation cost when you come for the interview, you will not be paid for the lost time and transportation. The location of your house will be captured by geographic information system (GIS) but this is only done to confirm that the

interviewer visited your house for the interview. Since the GIS reading doesn’t tell whose house is that house, your house will not be easily known by people who access the data given by you. On top of that only authorized people will have access to the data.

What will happen to the information about you?

The data that are registered about you will only be used in accordance with the purpose of the study as described above. All the data will be processed without name, but we will use a code number that links you to your data.

Only authorised project personnel will have access to the data. The data will be stored by the ABH Services PLC and USAID/CTB Ethiopia. It will not be possible to identify you when the results are published.

Voluntary participation

Participation in this survey is voluntary. You can choose not to answer any individual question or totally refuse to participate in the study. This will not have any consequences on you. However, we hope that you will participate fully in this survey since your views are very important.

If you wish to participate, please inform the data collector so that he/she can put a mark on the declaration of consent sheet which is found on the final page. If you have questions concerning the study, you may contact 1) Dr. Markos Feleke, CEO of ABH Services PLC (Phone Number: 251-911511610, email address: markos@abhethiopia.com) and 2) Dr. Eskindir Loha, the principal investigator of the study (Phone: 251-913 23 32 94, email address: eskindir_loha@yahoo.com).

CONSENT FOR PARTICIPATION IN THE TB KAP STUDY

I confirm that I have received the necessary information about the study and am willing to participate in the study.

Name of the participant: _______________________________________

Check box to mark the participant has given his/her verbal consent ☐

Date verbal consent given:____

Name of data collector who received verbal consent:_____________________________________

Signature of data collector who received verbal consent: __________________________________
